# Unique microbial diversity, community composition, and networks among Pacific Islander endocervical and vaginal microbiomes with and without *Chlamydia trachomatis* infection in Fiji

**DOI:** 10.1128/mbio.03063-23

**Published:** 2023-12-20

**Authors:** Sankhya Bommana, Yi-Juan Hu, Mike Kama, Ruohong Wang, Reshma Kodimerla, Kenan Jijakli, Timothy D. Read, Deborah Dean

**Affiliations:** 1 Department of Pediatrics, University of California San Francisco, Oakland, California, USA; 2 Department of Biostatistics and Bioinformatics, Emory University, Atlanta, Georgia, USA; 3 Ministry of Health and Medical Services, Suva, Fiji; 4 Department of Medicine, Emory University School of Medicine, Atlanta, Georgia, USA; 5 Department of Medicine, University of California San Francisco, San Francisco, California, USA; 6 Department of Bioengineering, Joint Graduate Program, University of California San Francisco and University of California Berkeley, San Francisco, California, USA; 7 Bixby Center for Global Reproductive Health, University of California San Francisco, San Francisco, California, USA; 8 University of California San Francisco, Benioff Center for Microbiome Medicine, San Francisco, California, USA; The Ohio State University School of Medicine, Columbus, Ohio, USA

**Keywords:** Pacific Islanders, *Chlamydia trachomatis*, metagenomic shotgun sequencing, pathogenesis, microbiome networks

## Abstract

Pacific Islanders (PI) are a vulnerable population with a high prevalence of *Chlamydia trachomatis* (*Ct*) sexually transmitted infections and remain underrepresented in research. Here, 258 vaginal and 92 paired endocervical samples from women of diverse ethnicities in Fiji were analyzed using metagenomic shotgun sequencing. Using VALENCIA, a sub-community state type (subCST) classifier, 93 (36.04%) vaginal and 38 (41.30%) endocervical microbiomes did not match reference subCSTs. We, therefore, designed four unique subCSTs, IV-D0, IV-D1, IV-D2, and IV-E—differentially dominated by *Gardnerella vaginalis*, *Prevotella* spp., and *Lactobacillus iners*—to improve microbiome classifications. *Ct*-infected paired endocervical and vaginal microbiomes were significantly more likely to have differential species relative abundance (81.58%) than uninfected pairs (35.84%; adjusted odds ratio, 7.93; 95% confidence interval, 2.93–21.93; *P* < 0.0001). Among paired microbiomes, the endocervix had a significantly higher alpha diversity and independently a higher alpha diversity for high-risk human papilloma virus (HPV) types compared to low-risk and no HPV. The *Ct-*infected endocervical network compared to all others had smaller clusters—indicating lower species abundance and decreased weight of interactions among bacterial groups within clusters—and no interactions with potentially beneficial *Lactobacillus* spp. These data suggest a distinct influence on this network from *Ct*-infected endocervical cells. Furthermore, the high relative abundance of *G. vaginalis* and *Prevotella* spp. may generate polymicrobial biofilms that predispose to and/or promote *Ct* and possibly HPV persistence and pathogenicity. While these data expand on the existing repertoire of endocervical and vaginal microbiomes and fill in knowledge gaps regarding PIs, prospective studies are needed to define causal associations to develop successful interventions.

**IMPORTANCE:**

*Chlamydia trachomatis* (*Ct*) is the most common sexually transmitted bacterium globally. Endocervical and vaginal microbiome interactions are rarely examined within the context of *Ct* or among vulnerable populations. We evaluated 258 vaginal and 92 paired endocervical samples from Fijian women using metagenomic shotgun sequencing. Over 37% of the microbiomes could not be classified into sub-community state types (subCSTs). We, therefore, developed subCSTs IV-D0, IV-D1, IV-D2, and IV-E—dominated primarily by *Gardnerella vaginalis*—to improve classification. Among paired microbiomes, the endocervix had a significantly higher alpha diversity and, independently, higher diversity for high-risk human papilloma virus (HPV) genotypes compared to low-risk and no HPV. *Ct-*infected endocervical networks had smaller clusters without interactions with potentially beneficial *Lactobacillus* spp. Overall, these data suggest that *G. vaginalis* may generate polymicrobial biofilms that predispose to and/or promote *Ct* and possibly HPV persistence and pathogenicity. Our findings expand on the existing repertoire of endocervical and vaginal microbiomes and fill in knowledge gaps regarding Pacific Islanders.

## INTRODUCTION

*Chlamydia trachomatis* (*Ct*) is the most common bacterial cause of sexually transmitted infections (STIs) worldwide. The World Health Organization estimates that over 130 million global cases occur each year (1, 2). The distribution of these infections is greatest in Africa and the Western Pacific Region (WPR) where there are about 61 million cases (1). The prevalence of *Ct* STIs ranges from 30% to 44% among teens and young adults in the Pacific Island Countries and Territories (PICT) of the WPR (3–6). Here, the combination of asymptomatic, untreated infection and syndromic management—treatment based on signs and symptoms as a proxy for infection—can lead to life-threatening ectopic pregnancy, infertility, and an increased risk of cervical cancer and HIV (7–10). Indeed, PICTs have the third highest prevalence of infertility in the world today (11).

The vaginal microbiome is influenced by resident microbes but also those associated with bacterial vaginosis (BV) and STIs, including *Ct*, *Neisseria gonorrhoeae* (*Ng*), *Trichomonas vaginalis* (*Tv*), *Mycoplasma genitalium* (*Mg*), HIV, and human papilloma virus (HPV) (12–14). Community state type I (CST-I), CST-II, CST-III, CST IV, and CST-V have been developed to classify vaginal microbiomes (15). CSTs I–III and V are dominated by *Lactobacillus crispatus*, *Lactobacillus gasseri*, *Lactobacillus iners*, or *Lactobacillus jensenni* that maintain vaginal homeostasis by secreting lactic acid, bacteriocins, and antimicrobial compounds, but not hydrogen peroxide, to inhibit anaerobic bacterial growth and protect against BV and STIs (16). CST-IV is deficient in *Lactobacillus* spp. and rich in anaerobic bacteria such as *Prevotella*, *Dialister*, *Atopobium*, *Gardnerella*, *Megasphaera*, *Peptoniphilus*, *Sneathia*, *Eggerthella*, *Aerococcus*, *Finegoldia*, and *Mobiluncus* genera. A diverse polymicrobial microbiome can increase the risk of *Ct* infection and other STIs (17, 18). Vaginal microbiome data from European, Asian, African, and Hispanic populations have distinct CSTs among these racial, ethnic, and geographic groups regardless of STI status (19–23). However, data from other ethnic and racial populations such as indigenous Australian, Native American, and Pacific Islander (PI) populations are lacking.

To provide insights into the epidemiology, structure, and function of the vaginal microbiome, the vaginal non-redundant gene catalog (VIRGO) (24) and sub-community state type (subCST) classifier VALENCIA (25) were developed. VIRGO was constructed from 264 vaginal metagenomes and 308 draft genomes of urogenital bacterial isolates from North American women and tested using 91 vaginal metagenomes from North American, African, and Chinese women. These data provided over 95% microbiome coverage applicable to these populations. VALENCIA classifies microbiomes into 13 subCSTs based on their similarity to a set of reference centroids built using a set of 13,160 16S rRNA sequencing-based taxonomic profiles from 1,975 North American, Black, White, Hispanic, and Asian women. It was validated using data sets of reproductive age eastern and southern African women and adolescent girls and ethnically and geographically diverse samples of postmenopausal women, although the number of women in each data set was not available.

We recently implemented VIRGO and VALENCIA on paired endocervical and vaginal metagenomes of PIs in Fiji as a pilot study, which involved age matching five *Ct*-infected women to those without *Ct* (26). VALENCIA could not accurately classify 4 of 10 vaginal and 4 of 10 endocervical microbiomes. Furthermore, because VIRGO does not contain viral, fungal, or protozoan pathogen sequences, such as HPV, *Candida*, and *T. vaginalis*, respectively, analyses for these pathogens were limited. We found that the microbiomes were largely dominated by species other than *Lactobacillus* regardless of STI status, and while endocervical and vaginal microbiomes were closely related, they were distinct in taxonomic composition, subCSTs, and metabolic function. However, our study was relatively small, and only ethnic iTaukei PIs were studied.

There is an obvious need to expand metagenome studies of endocervical and vaginal microbiomes from diverse ethnic and global populations to better understand microbial diversity, compositions, and networks that drive pathogenesis. Building on these knowledge gaps, we expanded our data set to 258 vaginal and 92 paired endocervical samples using metagenomic shotgun sequencing (MSS) to characterize vaginal and endocervical microbial relative abundance, composition, and network interactions for the diversity of ethnicities among women residing in Fiji. We also explored differences between paired microbiomes with and without the presence of *Ct*, other STIs, *Candida*, and BV.

## RESULTS

### Characteristics of study subjects, infection prevalence, and quality statistics of endocervical and vaginal MSS

Women 18 to 40 years of age attending Fiji Ministry of Health and Medical Services (MoHMS) health centers were enrolled in the parent study as described (3). The ethnicities in Fiji include ~57% iTaukei and ~37% Indo-Fijians (27, 28). Other ethnic groups include Chinese, European, mixed race, other PIs—IKiribati, Banabans, Tuvaluans, Tongans, Samoans, and Wallisians—and expatriates of various nationalities (28).

From the parent study, 258 vaginal samples, of which 92 had paired endocervical samples, were available. Paired samples were collected at the same time point from the same woman. Data on participants’ age, ethnicity, BV status, and infection status for *Ct*, *Ng*, *Tv*, HPV, and *Candida* were provided (Table S1). Age above 24 years and PI ethnicities were significantly associated with *Ct* infection (Table 1).

**TABLE 1:** Bivariate analysis for age group and ethnicity associated with *C. trachomatis* infection for study participants^*c*^

Variables	*N* ^*a*^	Endocervix (*n* = 46)				*N* ^*a*^	Vagina (*n* = 111)			
		*Ct*	No *Ct*	*P*-value	OR (95% CI)		*Ct*	No *Ct*	*P*-value^*d*^	OR (95% CI)
Age (years)	46	22	24			111	60	51		
18–24	15	4	11	–	ref	37	14	23	–	ref
25–40	31	18	13	0.0520	3.81 (1.05, 16.3)	74	46	28	**0.0168**	2.70 (1.21, 6.21)
Ethnicity^*b*^	46	22	24			111	60	51		
Indo-Fijian	11	1	10	–	ref	28	4	24	–	ref
iTaukei	26	16	10	**0.0136**	16.0 (2.50, 317)	64	45	19	**1.17e−05**	14.2 (4.74, 53.5)
Other PI	4	3	1	**0.0292**	30.0 (1.98, 1283)	10	7	3	**0.0026**	14.0 (2.76, 91.8)
Other	4	1	3	0.4402	3.33 (0.109, 104)	8	3	5	0.158462	3.60 (0.568, 22.2)
Missing	1	1	0	–	–	1	1	0	–	–

^*a*^ The total number of women in the study was 111; 46 endocervical samples were obtained from these women at the same time point as the vaginal samples, which we consider paired samples; paired endocervical and vaginal samples were also available at a second time point for the same 46 women, for a total of 350 samples (92 endocervical and vaginal samples paired from one time point + 92 paired from a second time point + 166 unpaired vaginal samples from the remaining women who did not have paired endocervical and vaginal samples).

^*b*^ Other PI ethnicity: IKiribati, Banabans, Tuvaluans, Tongans, Samoans, and Wallisians; other ethnicity: Chinese, European, and mixed race.

^*c*^ *Ct*, *C. trachomatis* infection; ref, reference for calculating *P*-value and OR, OR, odds ratio; CI, confidence interval; PI, Pacific Islander.

^*d*^ *P*-values that have been highlighted in bold are those that are statistically significant for that variable.

Results for BV using Amsel criteria (29) and for *Tv* and *Candida*—detected by wet prep on vaginal samples (provided by the parent study) and MetaPhlAn v3.0 (this study) for both vagina and endocervix—are shown in Table S1. Using multivariate logistic regression, there was a significant association with *Ct* for *Ng* in the endocervix and vagina; for high-risk (hr) HPV, *Tv*, and BV in the vagina; and for *Mg* in the endocervix, although the numbers were small (Table 2).

**TABLE 2:** Bivariate analysis and multivariate model for sexually transmitted infections, including HPV, *M. genitalium*, *N. gonorrhoeae*, and *T. vaginalis*, in addition to BV and their association with *Ct* infection for endocervical and vaginal samples^*b*^

Variables	*N* ^*a*^	Endocervix (*n* = 92)						*N* ^*a*^	Vagina (*n* = 258)					
		*Ct*	No *Ct*	*P*-value	OR (95% CI)	*P*-value	AOR (95% CI)		*Ct*	No *Ct*	*P*-value	OR (95% CI)	*P*-value^*c*^	AOR (95% CI)
HPV	92	40	52					258	99	159				
No infection	38	13	25	–	ref	–	–	101	33	68	–	ref	–	ref
lrHPV	29	15	14	0.152	2.06 (0.771, 5.64)	–	–	116	45	71	0.349	1.31 (0.748, 2.29)	–	–
hrHPV	25	12	13	0.276	1.78 (0.633, 5.05)	–	–	41	21	20	**0.0410**	2.16 (1.03,4.57)	**0.0329**	2.45 (1.08, 5.66)
Missing	0	0	0	–	–	–	–	0	0	0	–	–	–	–
*M. genitalium*	92	40	52					258	99	159				
Absent	85	34	51	–	ref	–	ref	249	95	154	–	ref	–	–
Present	7	6	1	**0.0463**	9.00 (1.45, 174)	0.0572	10.7 (1.35, 255)	9	4	5	0.7040	1.30 (0.314, 5.02)	–	–
Missing	0	0	0	–	–	–	–	0	0	0	–	–	–	–
*N. gonorrhoeae*	92	40	52					258	99	159				
Absent	53	15	38	–	ref	–	ref	197	67	130	–	ref	–	ref
Present	39	25	14	**0.0008**	4.52 (1.90, 11.3)	**0.0132**	3.49 (1.32, 9.70)	61	32	29	**0.0104**	2.14 (1.20, 3.85)	**0.0410**	1.94 (1.03, 3.68)
Missing	0	0	0	–	–	–	–	0	0	0	–	–	–	–
*T. vaginalis*	92	40	52					258	99	159				
Absent	86	37	49	–	ref	–	–	232	84	148	–	ref	–	ref
Present	6	3	3	0.740	1.32 (0.234, 7.51)	–	–	26	15	11	**0.0368**	2.40 (1.06, 5.60)	0.282	1.60 (0.685, 3.85)
Missing	0	0	0	–	–	–	–	0	0	0	–	–		
Bacterial vaginosis								258	99	159				
Absent	–	–	–	–	–	–	–	174	58	116	–	ref	–	ref
Present	–	–	–	–	–	–	–	39	24	15	**0.00150**	3.20 (1.58, 6.68)	**0.0153**	2.64 (1.22, 5.93)
Missing	–	–	–	–	–	–	–	45	17	28	–	–	–	–

^*a*^ The total number of women in the study was 111; 46 endocervical samples were obtained from these women at the same time point as 46 vaginal samples; paired endocervical and vaginal samples were available at a second time point for the same 46 women, which we consider paired samples, for a total of 350 samples (92 endocervical and vaginal samples paired from one time point + 92 endocervical and vaginal samples paired from a second time point + 166 unpaired vaginal samples from the remaining women who did not have paired endocervical and vaginal samples) for HPV, *M. genitalium*, *T. vaginalis*, and *N. gonorrhoeae* associations. The paired and unpaired vaginal samples were used in the BV analyses. Note that BV cannot be diagnosed in the endocervix.

^*b*^ *Ct*, *C. trachomatis* infection; ref, reference for calculating *P*-value and OR; OR, odds ratio; CI, confidence interval; PI, Pacific Islander; AOR, adjusted OR; HPV, human papilloma virus; hrHPV, high-risk HPV; lrHPV, low-risk HPV.

^*c*^ *P*-values that have been highlighted in bold are those that are statistically significant for that variable.

The 258 vaginal and 92 endocervical metagenomes yielded a total of ~26 billion and 9.5 billion raw reads of which 22.8 billion (87.36%) and 8.76 billion (91.4%) were identified as human contamination, respectively (Table S2).

Based on VIRGO, *Mg* reads were detected in 9 (3.5%) of 258 vaginal and 7 (7.6%) of 92 endocervical metagenomes (see Materials and Methods; Table S3). HPViewer (30) (Table S3) identified HPV in 157 (60.9%) of 258 vaginal and 54 (58.7%) of 92 endocervical samples. hrHPV types were found in 92 (35.66%) and 38 (41.30%) samples, respectively; not all paired samples had the same HPV type(s). The most common low-risk (lr) HPV types were 57 (*n* = 45; 25 vaginal, 20 endocervical) followed by HPV 62 (*n* = 42; 32 vaginal, 10 endocervical) and 90 (*n* = 40; 26 vaginal, 14 endocervical). The most common hrHPV types were 52 (*n* = 35; 24 vaginal, 11 endocervical) and 39 (*n* = 30; 17 vaginal, 13 endocervical). hrHPV types 16 and 18—responsible for most HPV-related cancers—were found in 24 (9.30%) of 258 vaginal and 11 (11.96%) of 92 endocervical samples and 18 (6.98%) vaginal and 21 (22.83%) endocervical samples, respectively. For *Tv*, six samples were positive by MSS alone in the endocervix and 22 in the vagina—10 by wet prep, 4 by wet prep and MSS, and 8 by MSS alone (Table S1). Few samples were positive for *Candida* in the vagina, and none, in the endocervix (Table S3).

### Expansion of existing CST classification system to include new subCSTs unique to endocervical and vaginal microbiomes of PIs

The microbiomes resolved into five CSTs (15) and 13 subCSTs based on VALENCIA, a nearest-centroid algorithm (25). The algorithm assigns a similarity score of 0.0 (no shared taxa) to 1.0 (all taxa shared at similar relative abundance) of the microbial profile of the sample to 1 of 13 reference centroids. In our previous (26) and current studies, several samples had low scores (<0.1–0.25) but also scores above 0.4, ranging from 0.019 to 0.808, where there was a discrepancy between microbiome species abundance and archetypal profile of the closest centroid. We, therefore, manually compared microbial relative abundance profiles of each microbiome with that of the assigned subCST, re-classifying samples regardless of their similarity score.

**Fig 1:**
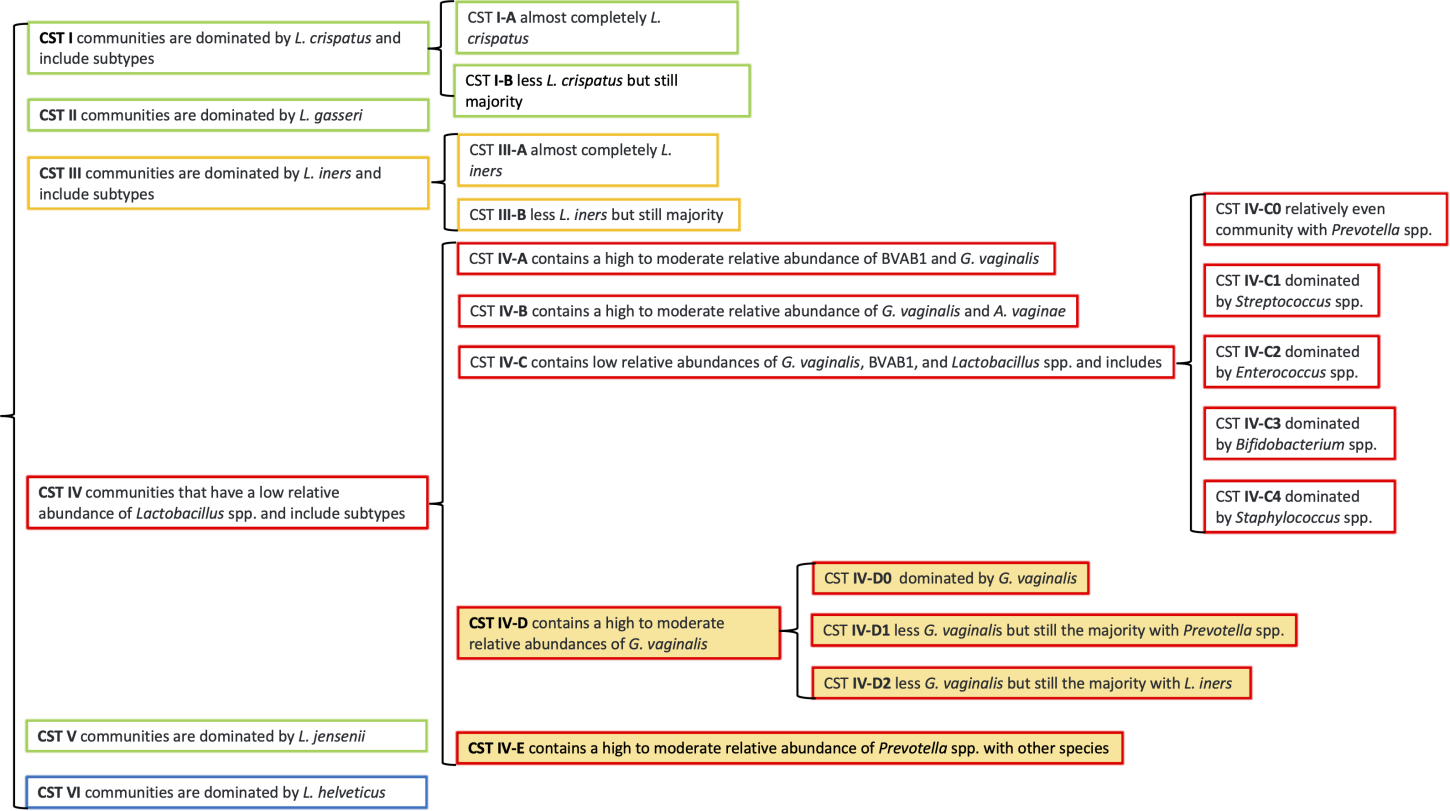
Classification of the endocervical and vaginal CSTs by VALENCIA with inclusion of four new subCSTs (IV-D0, IV-D1, IV-D2, and IV-E) identified among PI residing in Fiji. The new subCSTs (denoted in orange background) were constructed by averaging the relative abundance of all microbiota for the samples that fit the new subCST designation (*n* = 112; see Materials and Methods). A new CST VI suggested by Li et al. (31) is also included in the figure (blue outlined box).

The relative abundance profiles for 93 (36.04%) of 258 vaginal and 38 (41.3%) of 92 endocervical microbiomes did not match their assigned subCST (Table S4). These profiles indicated that they could be grouped into four new subCST categories within CST IV: IV-D0, dominated by *G. vaginalis* (*n* = 51); IV-D1, high to moderate relative abundance of *G. vaginalis* with *Prevotella* spp. (*n* = 18); IV-D2, high to moderate relative abundance of *G. vaginalis* with *L. iners* (*n* = 18); and IV-E, moderate *Prevotella* spp. with other species (*n* = 25) (Fig. 1, orange highlights). The new subCSTs were constructed by averaging the relative abundance of microbiota for the microbiomes (*n* = 131) that fit new subCST designation (Table S5).

**Fig 2:**
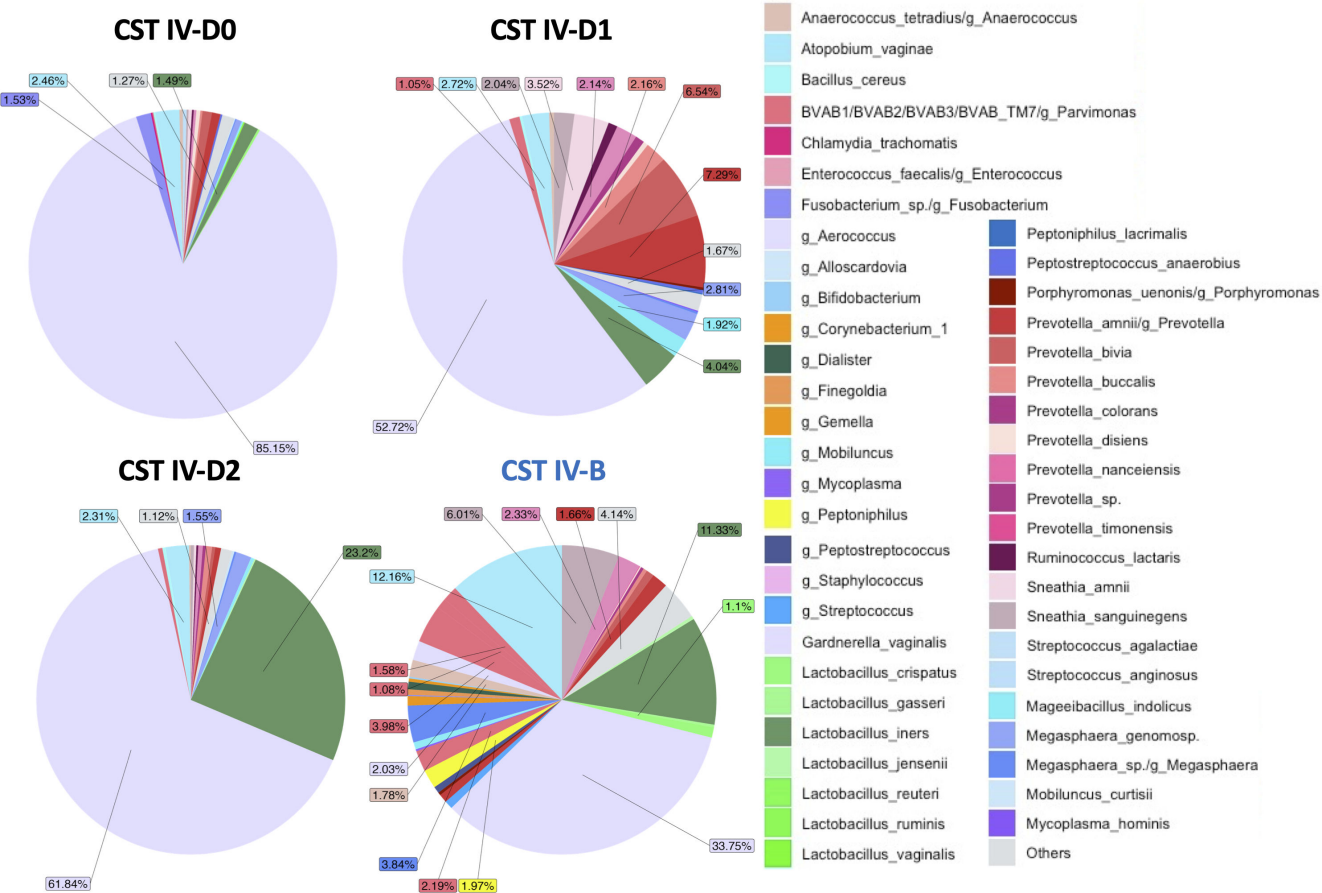
Pie chart representation of unique subCSTs IV-D0, IV-D1, and IV-D2 developed for endocervical and vaginal microbiomes from the Fiji metagenome database output from VIRGO. The microbiomes were originally assigned to subCST IV-B (right lower pie graph) using the VALENCIA classifier but were manually re-classified to the new subCSTs based on their relative abundance profiles that were not a good match with IV-B (see Materials and Methods). The new subCSTs were defined as IV-D0, dominated by *G. vaginalis;* IV-D1, less *G. vaginalis* but still the majority with *Prevotella* spp.; and IV-D2, less *G. vaginalis* but still the majority with *L. iners*. Relative abundance data were used to plot the pie charts of the new CSTs (Table S5).

Microbiomes classified as subCST IV-D0, IV-D1, and IV-D2 were originally assigned subCST IV-B by VALENCIA (Fig. 2). While subCST IV-B had a high to moderate relative abundance of *G. vaginalis* (33.75%) and *A. vaginae* (12.16%), the new subCSTs were different in composition. subCST IV-D0 had a higher relative abundance of *G. vaginalis* at 85.15% and lower abundance of *A. vaginae* at 2.46%. subCST IV-D1 had a higher relative abundance of both *G. vaginalis* (52.72%) and *Prevotella* spp. (20%), and subCST IV-D2 had a higher relative abundance of *G. vaginalis* (61.84%) and *L. iners* (23.2%) compared to CST IV-B (Fig. 2). Compared to more abundant bacteria and the diversity of species shown in the figures, individual species that were in relatively low abundance such as *Ct* are not visible.

**Fig 3:**
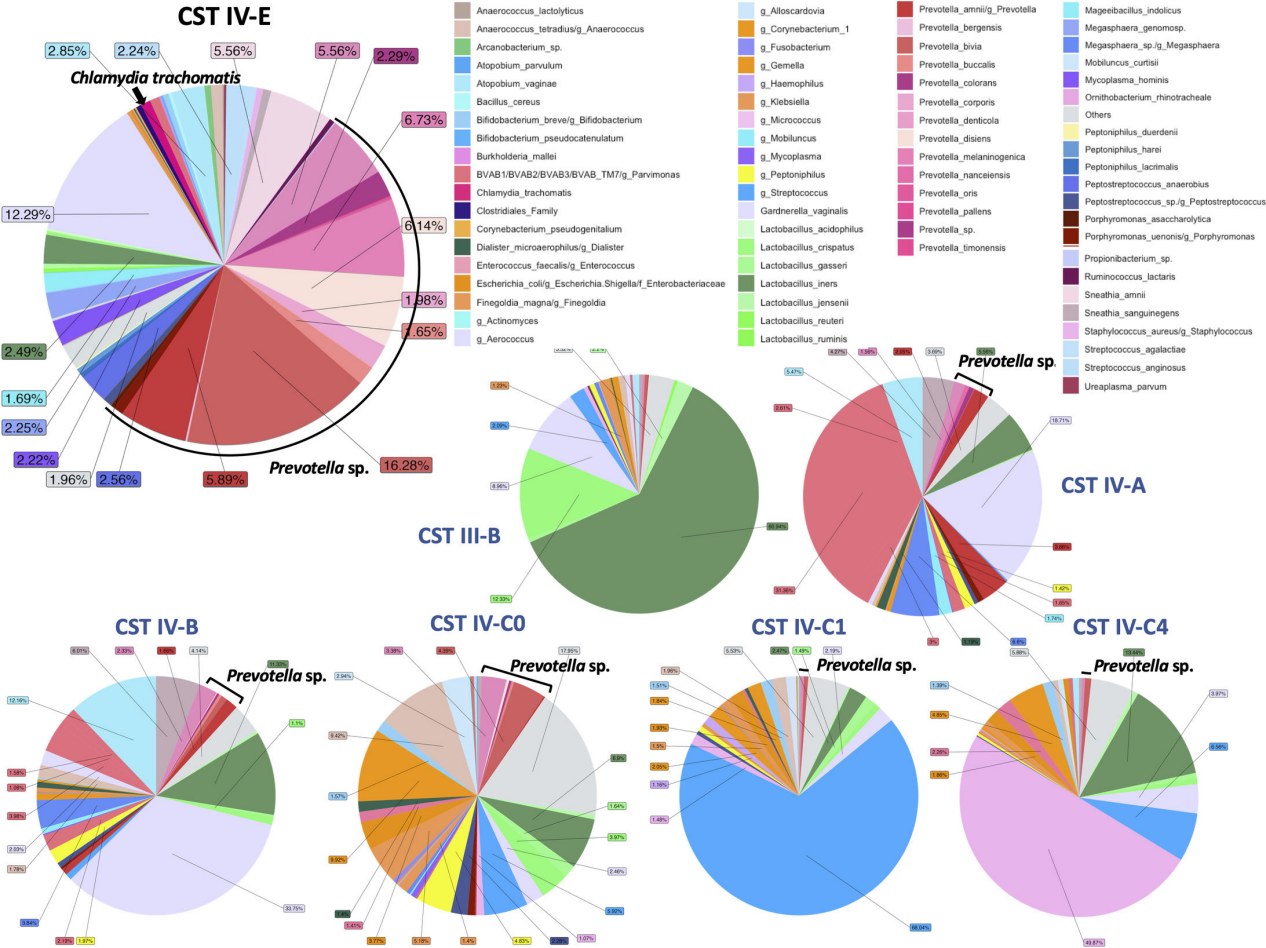
Pie chart representation of the unique subCST IV-E (upper left) developed for endocervical and vaginal microbiomes from the Fiji metagenome database output from VIRGO. The microbiomes were originally assigned to subCSTs III-B, IV-A, IV-B, IV-C0, IV-C1, and IV-C4 using the VALENCIA classifier (denoted by smaller pie charts and subCSTs in blue font) but were manually re-classified based on their relative abundance profile that was not a good match to the other subCSTs (see Materials and Methods). CST IV-E contains a high to moderate relative abundance of *Prevotella* spp. with other species. Relative abundance data were used to plot the subCST IV-E pie chart (Table S5).

Samples that were originally assigned as CST III-B, IV-A, IV-B, IV-C0, IV-C1, and IV-C4 by VALENCIA were re-classified as subCST IV-E (Fig. 3). CST IV-E had ~50% relative abundance of *Prevotella* spp. and 12.29% for *G. vaginalis*.

Of the 95 study participants whose microbiomes did not match the assigned subCSTs, iTaukei and other PI ethnicities (68/74 [91.89%]) were significantly more likely to not match compared to Indo-Fijian and other ethnicities (27/36 [75.00%]; adjusted odds ratio [AOR]: 3.78; 95% confidence interval [CI]: 1.23–11.64; *P* = 0.0205) (Table S4). Age group was not associated with a lack of matching to the assigned subCSTs.

### Within-host paired endocervical and vaginal species composition differs for women with and without *Ct* infection

**Fig 4:**
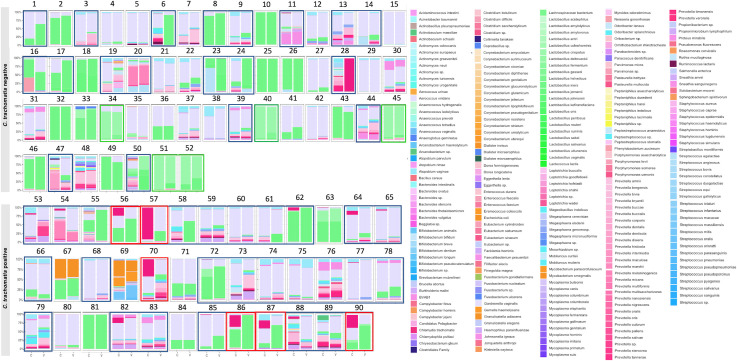
Relative abundance bar plots for *Ct*-infected and for uninfected paired endocervical and vaginal microbiomes. Only 90 pairs are shown due to lack of relative abundance data for two endocervical samples. Profiles in black boxes indicate paired endocervical and vaginal microbiomes with different microbial profiles. These microbiomes were dissimilar in species relative abundance for 19 (36.53%) of 52 *C. trachomatis* uninfected pairs and 31 (81.6%) of 38 *C. trachomatis* infected pairs. Green boxes show *L. crispatus*-dominant paired endocervical and vaginal microbiomes with similar profiles, which were only found in *C. trachomatis*-uninfected pairs (five [9.6%] of 52 pairs). Red boxes show moderate to high relative abundance for *C. trachomatis* endocervical microbiomes with dissimilar profiles, which were only found in *C. trachomatis*-infected pairs (six [15.79%] of 38 pairs). C, endocervix; V, vagina.

Figure 4 shows the relative abundance of taxa in paired vaginal and endocervical microbiomes based on *Ct* status. For *Ct-*uninfected endocervical and vaginal pairs, 19 (35.84%) of 53 pairs had differential species relative abundance between the anatomic sites, reflecting a difference in subCST (boxed in black, Fig. 4). The remaining 33 (63.46%) pairs had relatively similar species relative abundance. The presence of *L. crispatus*-dominated microbiomes in 5 (15.15%) of 33 similar pairs was unique to the uninfected group (boxed in green, Fig. 4). The majority of paired microbiomes in the uninfected group were dominated by *G. vaginalis* (23/52; 44.23%), *L. iners* (16/52; 30.76%), and *Prevotella* spp. (4/52; 7.69%).

*Ct*-infected paired microbiomes were statistically more likely to have differential species relative abundance (31/38 [81.57%]) between the two sites compared to *Ct*-uninfected pairs (19/53 [35.84%]; AOR: 7.93; 95% CI: 2.93–21.93; *P* < 0.0001) (boxed in black, Fig. 4). There was a higher relative abundance of *G. vaginalis* (18/38; 47.36%) compared to *L. iners* (7/38; 18.42%). Some profiles were dominated by *Prevotella* spp. (3/38; 7.89%) and *E. coli* (2/38; 5.26%). Interestingly, 6 (15.79%) of 38 pairs (subjects 56, 57, 70, 86, 87, and 90) had a moderate to high relative abundance of *Ct* in the endocervical but not vaginal microbiomes.

### Higher microbial diversity and species evenness were found in endocervical compared to vaginal microbiomes

**Fig 5:**
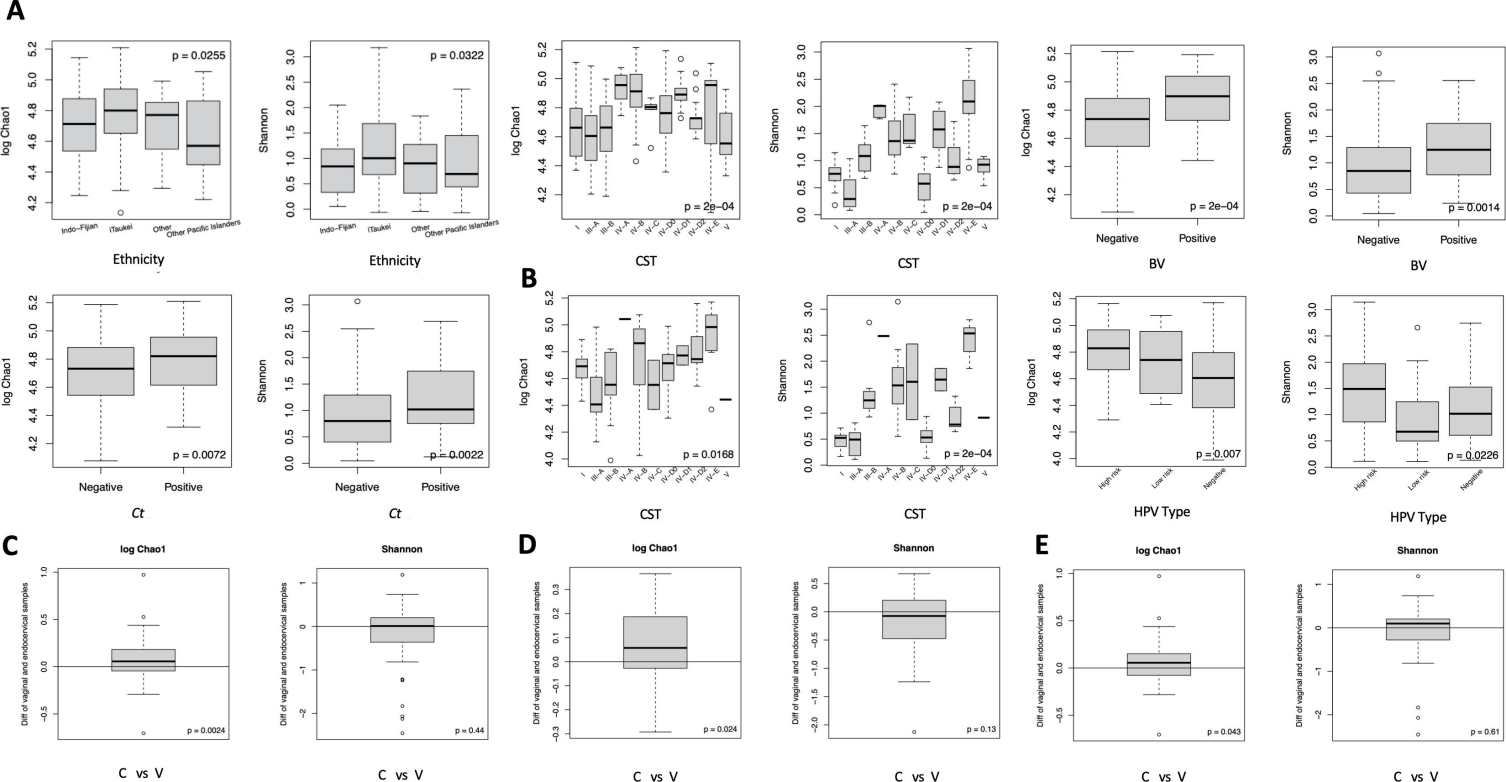
Alpha diversity metrics for the vaginal and endocervical microbiomes with categorical variables and estimated *P*-values based on the linear decomposition model (LDM; see Materials and Methods). (**A**) Shown in the four sets of bar plots for the vaginal microbiomes are the significantly higher alpha diversity (i.e., alpha diversity calculated by log Chao1 and Shannon) that was found when comparing groups within the categorical variables of ethnicity (i.e., Indo-Fijian, iTaukei, other PIs, other), CSTs, including subCSTs, BV status (i.e., present compared to not present), and *Ct* status (i.e., *Ct* infection compared to no infection). Groups within categorical variables that were not found to have a significantly higher alpha diversity are not shown. (**B**) Shown in the two sets of bar plots for the endocervical microbiomes are the significantly higher alpha diversity found when comparing groups within the categorical variables of CSTs (i.e., comparisons across all CSTs) and HPV types (i.e., comparing high-risk HPV, low-risk HPV, and no HPV; see Tables S1 and S3 for HPV risk types). HPV, human papilloma virus; hrHPV, high-risk HPV. (**C**) Shown in the set of bar plots for paired endocervical and vaginal microbiomes are the significantly higher alpha diversity found overall regardless of *Ct* infection (i.e., endocervical vs vaginal) and (**D**) independently for *C. trachomatis* infection (i.e., endocervical vs vaginal) and (**E**) for no *C. trachomatis* infection (i.e., endocervical vs vaginal). Log Chao1 and Shannon indices were both used to measure alpha diversity (see Materials and Methods). C, endocervix; V, vagina.

Alpha diversity was measured by Chao1 and Shannon index as shown in Fig. 5 using the linear decomposition model (LDM) (32). For vaginal microbiomes, iTaukei PIs had a significantly higher alpha diversity compared to Indo-Fijian and other ethnicities controlling for age and *Ct* (Chao1 *P* = 0.0255, Shannon *P* = 0.0322). Alpha diversity also varied significantly among subCSTs with higher diversity in subCST-IV (Chao1 *P* = 2e−04, Shannon *P* = 2e−04). Vaginal microbiomes with BV had higher diversity than those without BV controlling for *Ct* (Chao1 *P* = 2e−04, Shannon *P* = 0.0014), as did *Ct*-infected versus uninfected microbiomes (Chao1 *P* = 0.0072, Shannon *P* = 0.0022) controlling for ethnicity and age.

Endocervical microbiomes had a significantly higher alpha diversity for all subCST-IV classifications compared to other subCSTs (Chao *P* = 0.0168, Shannon *P* = 2e−04) (Fig. 5B). A higher alpha diversity was also found for hrHPV types compared to lrHPV types and no HPV infection (Chao *P* = 0.007, Shannon *P* = 0.0226).

Alpha diversity between paired vaginal and endocervical microbiomes differed significantly based on Chao1 (*P* = 0.0024) (Fig. 5C) that was influenced by the presence of *Ct* (Chao1: *P* = 0.024) (Fig. 5D) and less so by the absence of *Ct* (Chao1: *P* = 0.043) (Fig. 5E).

**Fig 6:**
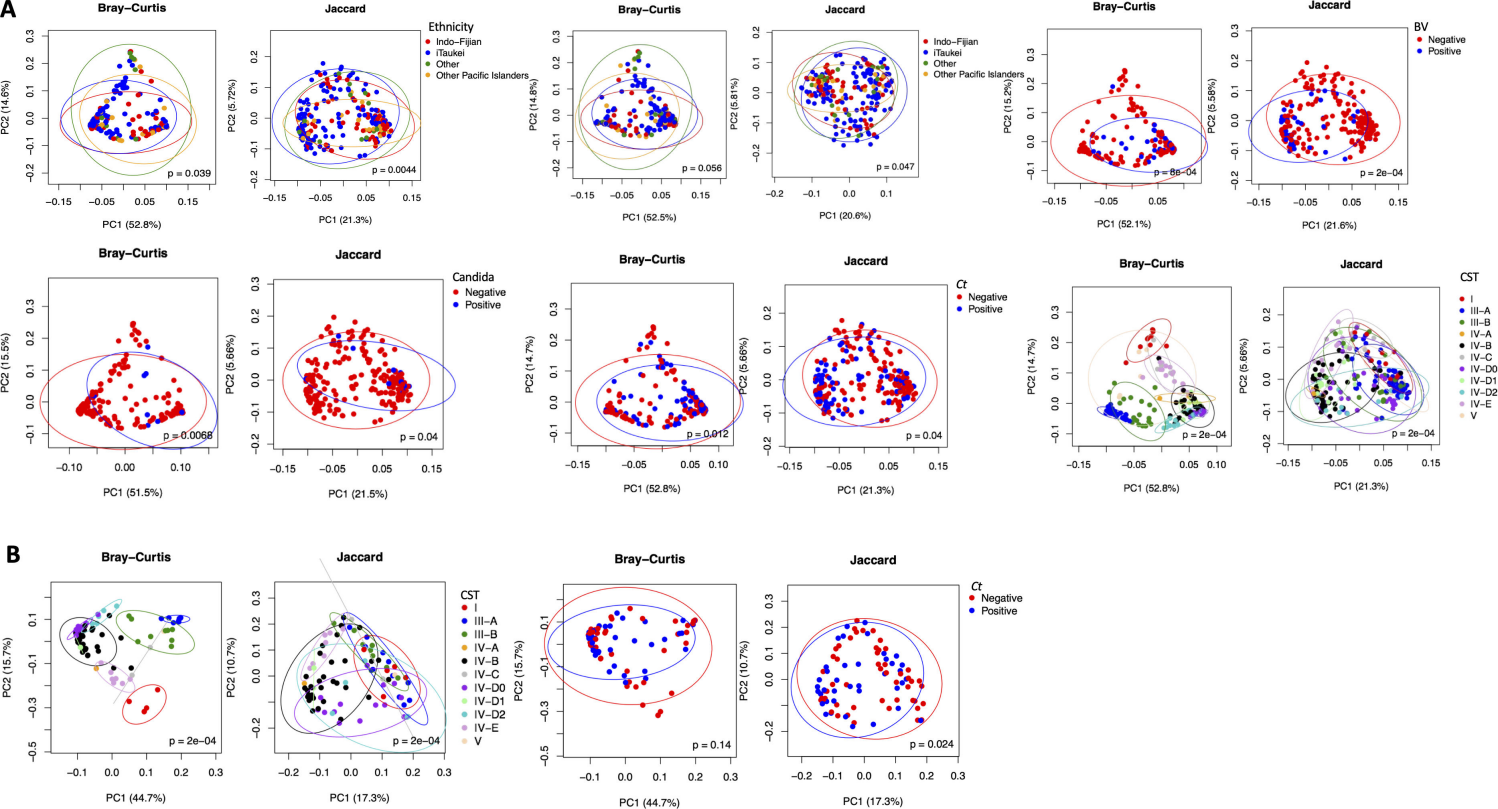
Beta diversity metrics for the vaginal and endocervical microbiomes with categorical variables and *P*-values estimated based on permutational multivariate analysis of variance (PERMANOVA). (**A**) Significant associations with the vaginal microbiomes are shown for ethnicity with and without controlling for *Ct* infection and age, CSTs, including subCSTs, BV, *Candida*, and *Ct* status. (**B**) Significant associations are shown for CSTs, including subCSTs, and *C. trachomatis* status with the endocervical microbiomes. Bray-Curtis and Jaccard indices were used to measure beta diversity (see Materials and Methods).

Divergence in microbial composition between samples was measured by beta diversity using Bray-Curtis and Jaccard metric. Principal component analysis (PCoA) showed significant segregation of vaginal microbiomes based on ethnic groups (*P* = 0.039 and *P* = 0.0044, respectively), subCST categories (*P* = 2e−04 and *P* = 2e−04), BV (*P* = 8e−04 and *P* = 2e−04), *Candida* (*P* = 0.0068 and *P* = 0.04), and *Ct* status (*P* = 0.012 and *P* = 0.04) (Fig. 6). In contrast, endocervical microbiomes were significantly segregated based on subCST-IV (*P* = 2e−04 and *P* = 2e−04) and *Ct* status (Jaccard only, *P* = 0.024) (Fig. 6).

### Ethnicity, BV, *Ct*, and anatomic site-specific associated species based on differential abundance and presence or absence of bacterial species

Based on LDM differential abundance analysis (32) of vaginal microbiomes, iTaukei and other PI ethnicities had a significantly higher abundance of pathogenic bacteria *S. amni*, *Leptotrichia goodfellowii*, and *Parvimonas* sp. compared to Indo-Fijian and other ethnic groups; *D. microaerophilus*, *Prevotella* sp., *Prevotella salivae*, and *Bifidobacterium animalis* were associated with iTaukei ethnicity only (Fig. S1). The high abundance of vaginal *L. crispatus* and *Actinomyces europaeus* was strongly associated with the “other” ethnic group. *Ruminococcus lactaris*, *M. indolicus*, *S. sanguinegens*, *Actinobaculum massiliae*, *Megashaera* sp., and *P. histicola* were enriched in endocervical microbiomes and significantly associated with iTaukei and/or other PI ethnicities, while *L. aciophilus*, *B. dentium*, and BVAB1 were associated with the other ethnicity (Fig. S2).

The 34 species significantly differentially abundant in BV-positive compared to BV-negative vaginal microbiomes included *Dialister*, *Leptotrichia*, *Mageeibacillus*, *Megasphaera*, *Mobiluncus*, *Mycoplasma*, *Parvimonas*, *Prevotella*, *Porphyromonas*, and *Sneathia* (Fig. S3). Based on presence or absence, 33 species were significantly higher in BV-positive microbiomes, overlapping with differential abundance data except for *L. crispatus* and *L. jensenii*, which were significantly higher in BV-negative microbiomes (Fig. S3).

Differentially abundant species significantly associated with *Ct-*infected vaginal microbiomes included *Prevotella* sp., *Tannerella forsythensis*, *L. goodfellowii*, *Lactobacillus pentosus*, *S. amnii*, and *Megasphaera* sp. (Fig. S4). For presence-absence data, *L. crispatus* was significantly higher in *Ct*-uninfected microbiomes, while *Sneathia sanguinegens*, *S. amnii*, *Tannerella forsythensis*, *Prevotella* sp., *Lactobacillus pentosus*, *Megasphaera micronuciformis*, *Mageeibacillus indolicus*, *Prevotella pallens*, *Prevotella amnii*, and *Bacteroides* sp. were significantly higher in *Ct-*infected microbiomes. No species were differentially abundant in *Ct*-infected endocervical microbiomes (data not shown.)

For *Ct-*infected paired microbiomes, 56 species were significantly differentially abundant in endocervical compared to vaginal microbiomes, and 12 species were differentially abundant in vaginal compared to endocervical biomes (Fig. S5). Based on species presence or absence, 71 species were significantly higher in endocervical versus vaginal biomes, and 19, in vaginal compared to endocervical biomes (Fig. S6).

For *Ct*-uninfected paired microbiomes, 26 species were significantly differentially abundant in vaginal compared to endocervical microbiomes, and six were differentially abundant in the endocervix (Fig. S7). For presence or absence data, 28 species were significantly higher in vaginal compared to endocervical microbiomes, and four, in the endocervix compared to the vagina (Fig. S8).

When considering anatomic site-specific differences regardless of *Ct* status, based on differential abundance, 67 species were significantly abundant in endocervical compared to paired vaginal microbiomes, and 18 species were significantly abundant in vaginal compared to endocervical microbiomes (Fig. S9). Based on presence or absence, 63 species were significantly higher in endocervical compared to paired vaginal microbiomes, and 12 species, in the vagina compared to the endocervix (Fig. S10).

### Paired endocervical and vaginal microbiomes show stable networks with anatomic site variation depending on *Ct* status

To understand bacterial community networks in vaginal and endocervical microbiomes, we employed Pearson’s correlation coefficient with a significance cutoff of *P* < 0.05 between different bacterial taxa in the respective microbiomes. Microbiota community clusters (i.e., nodes) within the networks were identified; modality was calculated to measure the degree of stability within densely connected communities in the network, which was greater than 0.8 for all networks, indicating high stability (33).

**Fig 7:**
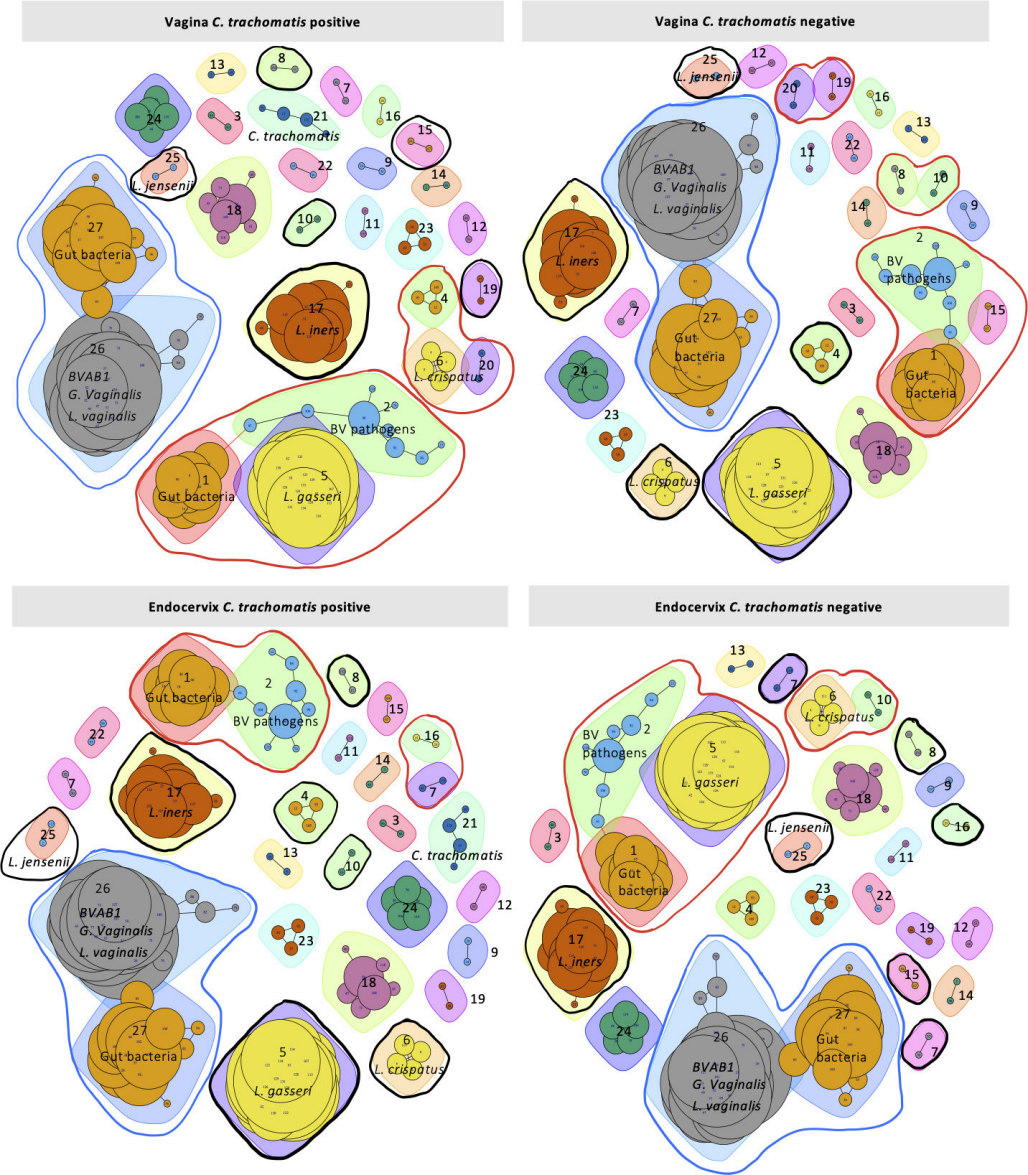
Network analysis based on Pearson’s correlation of the 92 *Ct*-infected or *Ct*-uninfected paired endocervical and vaginal microbiomes. Graph partitioning was performed to identify microbial community clusters within each network, and “modularity” was calculated to measure the degree of stability within the densely connected compartments or communities (see Materials and Methods). Nodes represent two or more groups—single or related species shown as different colored shapes—where the larger the size of the node, the greater the weight of the interactions of the groups within the network. Red lines show nodes with differential groups between paired endocervical and vaginal *C. trachomatis*-infected or *C. trachomatis*-uninfected networks and between endocervical and vaginal anatomic site networks. Black lines show differential independent groups between pairs and also between anatomic sites in the networks. Blue lines show similar nodes for all four *C. trachomatis*-infected and *C. trachomatis*-uninfected endocervical and vaginal networks.

Figure 7 shows numbered groups that represent a single species or related species; species within groups and clusters are listed in Table S6. Nodes are comprised of two or more groups, and their size denotes abundance of those species in the community, imbuing a greater weight of interactions for a node within the network (Fig. 7). The edges represent mutualistic relationships within species of the community (Fig. 7). Nine clusters were identified among *Ct-*infected and *Ct*-uninfected paired endocervical and vaginal microbiomes (Fig. 7). Cluster 1 consists of gut bacteria from groups 27 and 26. Cluster 1 areas were 35.60, 39.35, 36.94, and 37.04 for *Ct-*infected endocervical and vaginal and *Ct*-uninfected endocervical and vaginal microbiomes, respectively. Cluster 2 consists of group 1 gut bacteria and group 2 BV-associated bacteria. Cluster 3 comprises groups 1, 2, and 5; cluster 4, groups 1, 2, and 15; cluster 5, groups 4, 6, and 20; cluster 6, groups 2, 3, and 27; cluster 7, groups 19 and 20; cluster 8, groups 6 and 10; and cluster 9, groups 8 and 10.

## DISCUSSION

Major advances in our ability to analyze the microbial composition of vaginal microbiomes have revolutionized our understanding of the role they play in both health and disease. However, data on vulnerable populations from diverse ethnic and global populations are largely missing. Moreover, the relationship between endocervical and vaginal microbiomes has barely been examined. The former is of particular importance because the endocervix—not the vagina—is a primary site of infection for sexually transmitted *Ct*, *Ng*, and HPV. Here, we expanded on our pilot study (26) by evaluating 258 vaginal and 92 paired endocervical microbiomes for women of all ethnicities residing in Fiji. We characterized the microbial relative abundance, community composition, and networks of these microbiomes and examined the unique relationship between paired endocervical and vaginal microbiomes.

We previously found that women of iTaukei ethnicity were significantly more likely to have *Ct* than Indo-Fijians (3), consistent with the present study. Furthermore, there was a significantly higher microbial diversity for iTaukei ethnicity compared to Indo-Fijian, other PIs, and other ethnicities. Many BV-associated species such as *B. animalis*, *D. microaerophilus*, *Prevotella* sp., *P. salivae*, *S. amnii*, and *Parvimonas* sp. were also significantly associated with this ethnicity. Moreover, iTaukei and other PIs had a significantly diverse community composition of anaerobes compared to a predominance of *Lactobacillus* spp. such as *L. crispatus* for Indo-Fijian and other ethnicities. Such ethnicity- or race-based microbial clustering in microbiomes have also been reported for ethnic/racial subgroups of African descent and European/Caucasian background (21, 22).

Vaginal microbiomes with BV were significantly associated with *Ct* infection and also had higher microbial diversity with differences in community composition compared to those without BV, as has been reported (34, 35). Not surprisingly, BV-associated bacteria including *Megasphaera* spp., *Sneathia* spp., *G. vaginalis*, and *Prevotella* spp. were present at a significantly higher relative abundance compared to BV-negative microbiomes. *A. intestenii*, *T. forsythensis*, and *E. saburreum* were newly associated with BV. *A. intestenii* is an anaerobic intestinal commensal that has previously been found in the vaginal flora (36). The other species are present in oral biomes and linked to periodontal disease and/or respiratory infections (37–39). Their association with BV is not understood, although oral to genital autoinoculation or transfer during sex is a possibility.

Xpert CT/NG and wet prep tests on vaginal samples were able to detect *Ct*, *Ng*, *Tv*, and *Candida*, while MSS detected each along with *Mg*. In our previous study, *Mg* was found in 100% of samples using One Codex. In contrast, only 3.5% and 7.6% of vaginal and endocervical microbiomes had *Mg* using VIRGO (24). This discrepancy is not surprising, since complete *Mg* genomes were used to build the VIRGO database, which is therefore highly specific.

HPV types were identified using HPViewer (30), providing much needed data on the distribution of genotypes among PIs in the WPR. The only published study of HPV genotypes in PICTs identified hrHPV genotypes 16, 18, 33, 39, 45, and 52 among cervical cancer specimens along with lrHPV genotypes 54 and 67 that are infrequently found in these malignancies (40, 41); no data were available for women without cervical cancer. hrHPV 16, 18, 39, and 52 were also found in our Fijian population. Of the common lrHPV types present in the population, HPV 57 has been associated with oral papillomas (42). A possible oral to genital auto-inoculation or sexual transmission could explain the unexpected presence of this genotype in genital microbiomes.

Although no significant association of endocervical hrHPV genotypes with ethnicity, age, or *Ct* infection was found, not surprisingly, these high-risk types were associated with higher microbial diversity compared to low-risk types or no infection in the endocervical microbiome. This finding is consistent with two recent studies in China that showed a significantly increased alpha diversity in the HPV-infected cervix compared to those without HPV (43, 44).

Many endocervical (41.3%) and vaginal (36.04%) microbial relative abundance profiles did not match their assigned subCST based on VALENCIA. By manually comparing the species relative abundance of each microbiome with their assigned subCSTs, we developed four new subCSTs—IV-D0, IV-D1, IV-D2, and IV-E—that provide a more specific classification for Fijian microbiomes. Microbiomes classified as IV-B required reclassification as IV-D0, IV-D1, and IV-D2, and those assigned as CST III-B, IV-A, IV-B, IV-C0, IV-C1, or IV-C4 were re-classified as CST IV-E. The need to expand CST classifications is supported by a 2022 study of ethnic Han Chinese where a predominance of *L. helveticus* was discovered based on 16S rRNA sequencing of vaginal microbiomes not reflected in the VALENCIA reference centroids (31). They proposed a new CST-VI to represent communities that are dominated by this species. Additional CSTs will likely be required to further define endocervical and vaginal microbial landscapes, as more diverse and globally represented populations are studied.

The new subCSTs IV-D0, IV-D1, IV-D2, and IV-E had a predominance of *G. vaginalis*, *L. iners*, and *Prevotella* spp. along with other BV-associated pathogens. Many of these microbiomes may have a predisposition to *Ct* and/or HPV infections in this population. While there was no association between new subCSTs that did not match assigned subCSTs and *Ct* infection, there was a significant association with iTaukei and Other PI ethnicities.

Paired microbiomes had unique attributes depending on *Ct* status. Among *Ct-*uninfected pairs, while a little over a third had differential species relative abundance between anatomic sites, 15.15% of the pairs with similar species were dominated by *L. crispatus* with a slightly greater relative abundance in the endocervix. This is in contrast to a study where uninfected Chinese women were found to have a higher abundance of this species in the vagina compared to the endocervix (45). *L. crispatus* produces D-lactic acid and bacteriocins that may trap or inhibit pathogen attachment to host cells, protecting against STIs and also BV to maintain microbiome eubiosis (46–48). Similar to studies of South African adolescents and Italian women, we found that *L. crispatus*-dominated microbiomes were less likely to be associated with *Ct* (13, 49).

Among *Ct-*infected pairs, over 80% had differential species relative abundance with 72 species showing a significant difference between the two anatomic sites. Not surprisingly, these pairs were significantly more likely to have differential species relative abundance between sites compared to *Ct-*uninfected pairs. The *Ct*-infected endocervix also had a higher microbial diversity compared to the paired vaginal microbiome. These data are consistent with our previous study (26) and a study of adolescents where paired endocervical and vaginal microbiomes were also examined (13). A recent investigation of HPV infections found similar results (50). Additionally, the high dominance of *G. vaginalis* with other BV-associated pathogens such as *Prevotella*, *Megasphaera*, and *Atopobium* among *Ct-*infected microbiomes is supported by investigations in South Africa and Italy, although 16S rRNA-based sequencing was used instead of metagenomics (13, 49). The relatively lower dominance of *L. iners* (18.4%) compared to *Ct*-uninfected pairs(30.7%) could be explained by the fact that *L. iners* lacks the essential amino acid synthesis repertoire, forcing it to rely on exogenous host amino acids, which would make it more sensitive to community composition and perhaps less successful in competing with other microbes (51). However, *L. iners* has been shown in other studies to be associated with *Ct* infection and is more prevalent among women of African descent than other racial or ethnic groups (52–54).

Six (15.79%) of the *Ct-*infected endocervical microbiomes were dominated by a moderate to high abundance of *Ct*, but not in the paired vaginal microbiomes. Although the columnar epithelial cells of the endocervix are the target for *Ct* infection, this level of abundance has not previously been reported and was a unique occurrence in PIs. Indeed, few studies that evaluated the endocervical microbiome did not find this level of *Ct* abundance among, for example, Malaysian, Chinese, Indian, Italian, and South African women (13, 49, 55). One concern is that a high burden of *Ct* may contribute to upper genital tract infections and the high rate of infertility in Fiji (11, 56). A larger prospective study is required to assess the frequency of our findings in PI ethnicities and the significance in terms of microbial composition and disease pathogenesis, including fertility.

Nine clusters or nodes were identified in the paired endocervical and vaginal microbiome networks with shifts in size and composition depending on *Ct* status and anatomic site. Cluster 1, comprising a combination of gut bacteria and BV-associated species, was predominant in all networks. This finding reflects the very nature of PI microbiomes in that they tend to be highly diverse and rich in pathogenic bacteria regardless of *Ct* status. However, in the *Ct-*infected endocervical network, this cluster was appreciably smaller compared to all others, and no clusters included *Lactobacillus* spp. (i.e., groups 5, 6, 17, or 25); two of the three clusters in the *Ct-*infected vaginal microbiome contained *L crispatus* and *L. gasseri*. Since *Ct* infects the endocervical epithelium and is found either inside sloughed endocervical cells or loose in the vagina, the influence of *Ct* on each anatomic site might be distinct. Nodes containing *Lactobacillus* spp. may interact with BV-associated species and gut bacteria to dampen the effects of these pathogenic species or vice versa, and *Ct*-infected endocervical cells may actually work to dissociate or prevent *Lactobacillus* spp. interactions. This would be consistent with the smaller nodes that lack *Lactobacillus* spp. in the *Ct*-infected endocervical networks. In contrast, *Ct*-uninfected endocervical networks had much larger nodes where *L. gasseri* (group 5) was associated with gut (group 1) and BV-associated (group 2) bacteria. Our hypothesis may also explain the similarity between *Ct*-uninfected endocervical and *Ct*-infected vaginal networks where *Ct* does not infect the vaginal squamous epithelial cells. These network interactions are, therefore, likely driven by the microbial milieu of gut and BV-associated bacteria that are influenced by the actual *Ct* infection of cells in the endocervix and not by the simple presence of *Ct*, free or otherwise, as in the vagina. In addition, the high abundance of *G. vaginalis* may form biofilms but also interact with other BV-associated species to generate polymicrobial biofilms that increase the risk and persistence of *Ct* and other STIs such as HPV (57, 58). Additional studies will be needed to tease out these pathogenic interactions.

## MATERIALS AND METHODS

### Sample collection and patient characteristics

Women attending Fijian MoHMS health centers in the Central Division, Viti Levu, Fiji, were enrolled after informed consent as part of the parent study (3). Human subject’s approval was obtained from the institutional review boards of the Fijian MoHMS and the University of California San Francisco. Women of various ethnicities residing in Fiji aged 18 to 40 years were enrolled unless they had HIV, untreated syphilis, a diagnosis of cancer, or received antibiotics within the prior month. The parent study provided 258 de-identified vaginal and 92 paired endocervical samples collected from the same women at the same time point. All swabs were collected by trained clinicians. Vaginal samples were tested using the Xpert CT/NG assay (Cepheid); endocervical samples were screened for *Ct* using an in-house qPCR as described (26). Samples were additionally tested for *Tv* and *Candida* by wet prep. Amsel criteria were used to diagnose BV required three or more Amsel criteria (29).

### Sample processing, metagenomic shotgun sequencing, and taxonomic analysis

Genomic (g)DNA was extracted from swabs using a lysozyme cocktail prior to gDNA purification using the QIAmp DNA Mini Kit (Qiagen, Germantown, MD) as described (26). DNA concentrations were measured by the Qubit dsDNA assay kit (Invitrogen, Carlsbad, CA). MSS libraries were constructed using Illumina Nextera XT kits and sequenced using 150 nucleotide paired-end reads on an Illumina Novaseq PE150 platform.

Sequence data were processed using BBDuk v38.96 for Illumina adapter and quality trimming (59) to an average quality score of >10 and excised if scores were <10. Human read contaminants were removed using BBSplit v38.96 (59) with version 38 of the human genome (RefSeq: GCF_000001405.26). Ambiguous reads or reads not clearly human in origin were preserved for downstream processing. Data were then profiled using Metaphlan v3.0.14 for species identification (60, 61).

VIRGO (24) was run on post-processed microbial reads as described (26). The number of confirmed reads for each STI and *Candida* were divided by the total number of microbial reads for that sample and then multiplied by 1 million to arrive at a value for reads per million for that pathogen (62). HPViewer (30) was used to identify and quantify HPV genotypes in the metagenomes.

### CST assignments

The abundance data generated from VIRGO (24) was used for classification of vaginal and endocervical microbiomes into subCSTs using VALENCIA (25) based on the highest similarity score to 13 reference centroids. From our previous study (26) and a much larger sample size in the present study, there was a high discrepancy between microbiome species composition and subCST scores. We therefore manually compared VALENCIA assigned subCSTs with species composition of the 258 vaginal and 92 endocervical microbiomes to determine whether any new subCSTs could be developed.

### Statistical analysis and visualization

Microbiome analysis was performed using R version 4.0.3 including stats (4.0.3), dplyr (1.0.8) and broom (1.0.1) packages (63), Vegan (v.1.4–5) (64), Adespatial (v.0.3–14) (65), ggplot2 (v.3.3.5) (66), CGPfunctions (v.0.6.3) (67), FSA (v.0.9.3) (68), ggpubr (v.0.4.0) (69), and LDM (32). Bivariate and multivariate analyses of association between covariates and *Ct* were performed controlling for confounding variables. Results were expressed as odds ratios adjusted with 95% CI. Differences in log Chao1 and also Shannon indices between paired endocervical and vaginal microbiomes were estimated using odds ratio with 95% CI and tested by paired two-sample *t* test, χ^2^ test of association, or Fisher’s exact test when cells were small. Differences between *Ct-*infected and *Ct-*uninfected microbiomes were tested by unpaired two-sample *t* test. The relative abundance of the top 25 species within each patient’s vaginal and endocervical microbiome was visualized using the ggplot2 (v.3.3.5) library (66). PCoA was used to visualize clustering based on Bray-Curtis distances and Jaccard with statistical hypothesis testing performed by permutational multivariate analysis of variance (PERMANOVA) (see below). Statistical comparisons between *Ct-*infected and *Ct-*uninfected groups was performed using Kruskal-Wallis test and LDM.

Correlations between different bacterial taxa in the microbiomes based on *Ct* status were determined by Pearson’s correlation coefficients using R packages igraph (v. 1.3.0) (33), Hmisc (v. 4.6-0) (70), and Matrix (v. 1.4-1) (71). Graph partitioning of networks was performed to identify community clusters within each network, and “modularity” was calculated to measure the degree of stability within the densely connected communities (33).

Differential relative abundance association testing was performed using LDM, which is based on linear models of regressing individual taxon data on the covariate to test, controlling for confounding variables (32, 72). Taxon data are taken to be relative abundances, arcsin-root-transformed relative abundances, and presence-absence status, and the results from analyzing all data scales were combined to produce overall tests of differential abundance at both species and community levels. LDM bases the inference on a permutation procedure, which is important to preserve data structure and thus correlations in our data. The permutation procedure was performed by stratifying groups of subjects who each had the same number of visits. For each group of subjects, covariate values from a subject were shuffled as a set with the covariate values from another subject. Additionally, covariate values within a subject were also shuffled. The species-level analysis detected associated species at the nominal False discovery rate (FDR) level of 10%. The community-level analysis aggregated information from the species-level analysis. Both the global *P*-value and individual *P*-values in boxplots were produced by LDM. LDM for presence-absence analysis (72) is based on rarefaction, and LDM aggregates information from the all rarefied taxa count table. *P*-values for testing associations of each beta diversity metric with a variable was based on the PERMANOVA method (32, 73) using the same permutation procedure described above. *P*-values were adjusted for multiple testing by Sandve’s algorithm integrated in the LDM package (72).

## Data Availability

MSS reads are available as FASTQ files with associated metadata at the NCBI-SRA under BioProject ID PRJNA982400. Table S7 provides accession numbers for all samples in NCBI. All scripts on data analysis are available at https://github.com/ddeanlab/Microbial-PI-Ct-MSS.

## SUPPLEMENTAL MATERIAL

Supplemental Figures mbio.03063-23-s0001.pdf Figures S1 to S10.

Table S1 mbio.03063-23-s0002.xlsx Participant characteristics.

Table S2 mbio.03063-23-s0003.xlsx Metagenomic shotgun sequencing results and quality control statistics.

Table S3 mbio.03063-23-s0004.xlsx MSS data for *N. gonorrhoeae* and *M. genitalium* confirmed by VIRGO, *T. vaginalis* and *C. albicans* confirmed by MetaPhlAn v3.0, and HPV by confirmed HPViewer.

Table S4 mbio.03063-23-s0005.xlsx New classification of subCSTs for Pacific Islanders and metrics for age, ethnicity and *C. trachomatis* infection status.

Table S5 mbio.03063-23-s0006.xlsx Average of the relative abundance data for each species of the newly assigned sub community state types (subCSTs).

Table S6 mbio.03063-23-s0007.xlsx List of all species within the groups and clusters of vaginal and endocervical microbiome networks (see Fig. 7).

Table S7 mbio.03063-23-s0008.xlsx Accession numbers for all metagenomic samples submitted to NCBI.

ASM does not own the copyrights to Supplemental Material that may be linked to, or accessed through, an article. The authors have granted ASM a non-exclusive, world-wide license to publish the Supplemental Material files. Please contact the corresponding author directly for reuse.
