# Supplementary material for: Unique microbial diversity, community composition, and networks among Pacific Islander endocervical and vaginal microbiomes with and without Chlamydia trachomatis infection in Fiji
Source: mBio. 2023 Dec 20;15(1):e03063-23. doi: 10.1128/mbio.03063-23 (PMC10790706; doi:10.1128/mbio.03063-23)

Unique microbial diversity, community composition and networks among Pacific Islander endocervical and vaginal microbiomes with and without *Chlamydia trachomatis* infection in Fiji

Sankhya Bommana[a], Yi-Juan Hu[b], Mike Kama[c], Ruohong Wang[a], Reshma Kodimerla, Kenan Jijakli[d], Timothy D. Read[d*], Deborah Dean[a,e,f,g,h*]

[a]Department of Pediatrics, University of California San Francisco, Oakland, CA, USA
[b]Department of Biostatistics and Bioinformatics, Emory University, Atlanta, GA, USA
[c]Ministry of Health and Medical Services, Suva, Fiji
[d]Department of Medicine, Emory University School of Medicine, Atlanta, Georgia, USA
[e]Department of Medicine, University of California San Francisco, San Francisco, CA, USA
[f]Department of Bioengineering, Joint Graduate Program, University of California San Francisco and University of California Berkeley, San Francisco, CA, USA
[g]Bixby Center for Global Reproductive Health, University of California San Francisco, San Francisco, CA, USA
[h]Benioff Center for Microbiome Medicine, University of California San Francisco, San Francisco, CA, USA

SUPPLEMENTAL MATERIAL

Supplemental Figures

Supplementary Figure 1. Bacterial species significantly associated with iTaukei ethnicity based on differential abundance (A) or their presence or absence (B) for the vaginal microbiomes. Linear Decomposition Model (LDM) was used for statistical associations (see Methods).

Supplementary Figure 2. Bacterial species significantly associated with iTaukei ethnicity based on differential abundance (A) or their presence or absence (B) for the endocervical microbiomes. Linear Decomposition Model (LDM) was used for statistical associations (see Methods).

Supplementary Figure 3. Bacterial species significantly associated with presence of bacterial vaginosis (BV) for the vaginal microbiome based on differential abundance (A) or their presence or absence (B). Linear Decomposition Model (LDM) was used for statistical associations (see Methods). Note that BV cannot be measured for the endocervix.

Supplementary Figure 4. Bacterial species significantly associated with *C. trachomatis* infection (positive) compared to no infection (negative) for the vaginal microbiomes based on differential abundance (A) or their presence or absence (B). Linear Decomposition Model (LDM) was used for statistical associations (see Methods). No *C. trachomatis* associated species were identified for the endocervix.

Supplementary Figure 5. Bacterial species significantly associated with anatomic site (endocervix or vagina) based on differential abundance for the *C. trachomatis* infected paired endocervical

and vaginal microbiomes. Linear Decomposition Model (LDM) was used for statistical associations (see Methods). C, endocervix; V, vagina.

Supplementary Figure 6. Bacterial species significantly associated with anatomic site (endocervix or vagina) based on presence or absence for the *C. trachomatis* infected paired endocervical and vaginal microbiomes. Linear Decomposition Model (LDM) was used for statistical associations (see Methods). C, endocervix; V, vagina.

Supplementary Figure 7. Bacterial species significantly associated with anatomic site (endocervix or vagina) based on differential abundance for the *C. trachomatis* uninfected paired endocervical and vaginal microbiomes. Linear Decomposition Model (LDM) was used for statistical associations (see Methods). C, endocervix; V, vagina.

Supplementary Figure 8. Bacterial species significantly associated with anatomic site (endocervix or vagina) based on presence or absence for the *C. trachomatis* uninfected paired endocervical and vaginal microbiomes. Linear Decomposition Model (LDM) was used for statistical associations (see Methods). C, endocervix; V, vagina.

Supplementary Figure 9. Species significantly associated with anatomic site (endocervix or vagina) based on differential abundance for the paired endocervical and vaginal microbiomes regardless of *C. trachomatis* infection status. Linear Decomposition Model (LDM) was used for statistical associations (see Methods). C, endocervix; V, vagina.

Supplementary Figure 10. Species significantly associated with anatomic site (endocervix or vagina) based on presence or absence for the paired endocervical and vaginal microbiomes regardless of *C. trachomatis* infection status. Linear Decomposition Model (LDM) was used for statistical associations (see Methods). C, endocervix; V, vagina.

Supplementary Tables

Supplementary Table 1. Participant characteristics including infection with various sexually transmitted infections, bacterial vaginosis (BV) and *Candida*.

Supplementary Table 2. Metagenomic shotgun sequencing results and quality control statistics.

Supplementary Table 3. Metagenomic shotgun sequencing (MSS) data for *Neisseria gonorrhoeae* and *Mycoplasma genitalium* confirmed by VIRGO; *Trichomonas vaginalis* and *Candida albicans* confirmed by MetaPhlAn v3.0; and HPV by HPViewer.

Supplementary Table 4. New classification of subCSTs for Pacific Islanders, and metrics for age, ethnicity and *C. trachomatis* infection status.

Supplementary Table 5. Average of the relative abundance data for each species of the newly assigned sub community state types (subCSTs).

Supplementary Table 6. List of all species within the groups and clusters of vaginal and endocervical microbiome networks (see Figure 7).

Supplementary Table 7. Accession numbers for all metagenomic samples submitted to NCBI.

Supplementary Figure 1

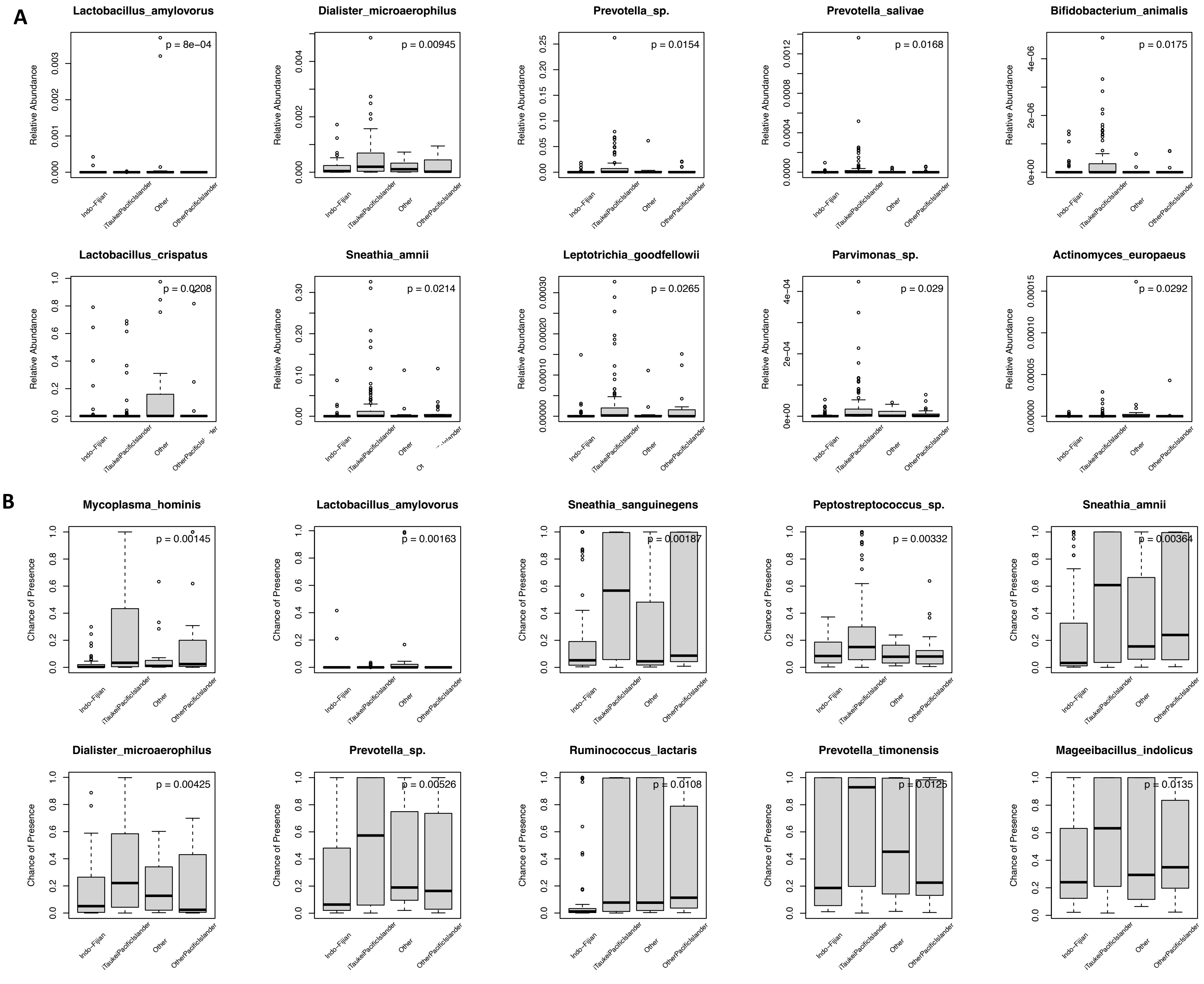

Supplementary Figure 2

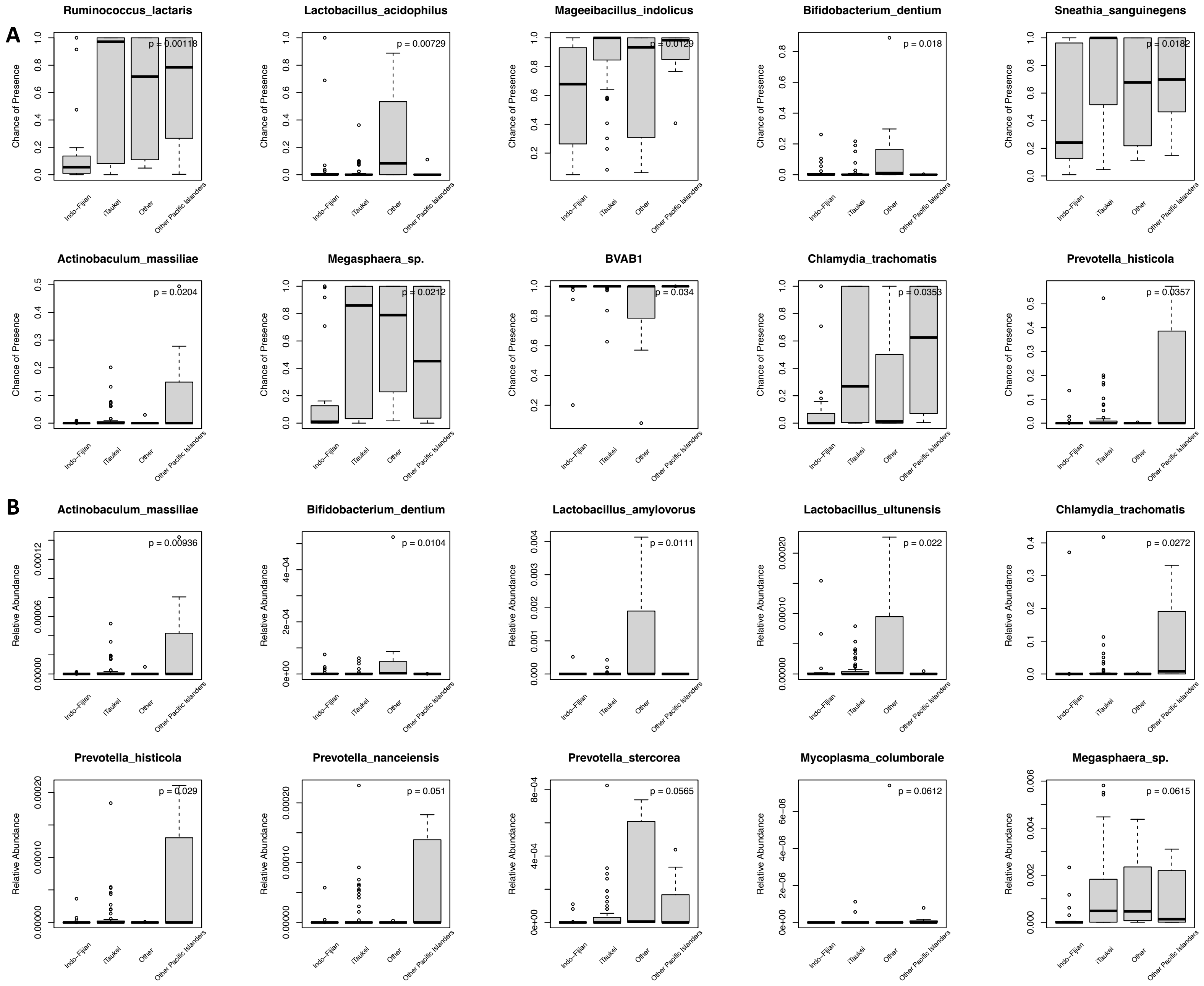

Supplementary Figure 3A

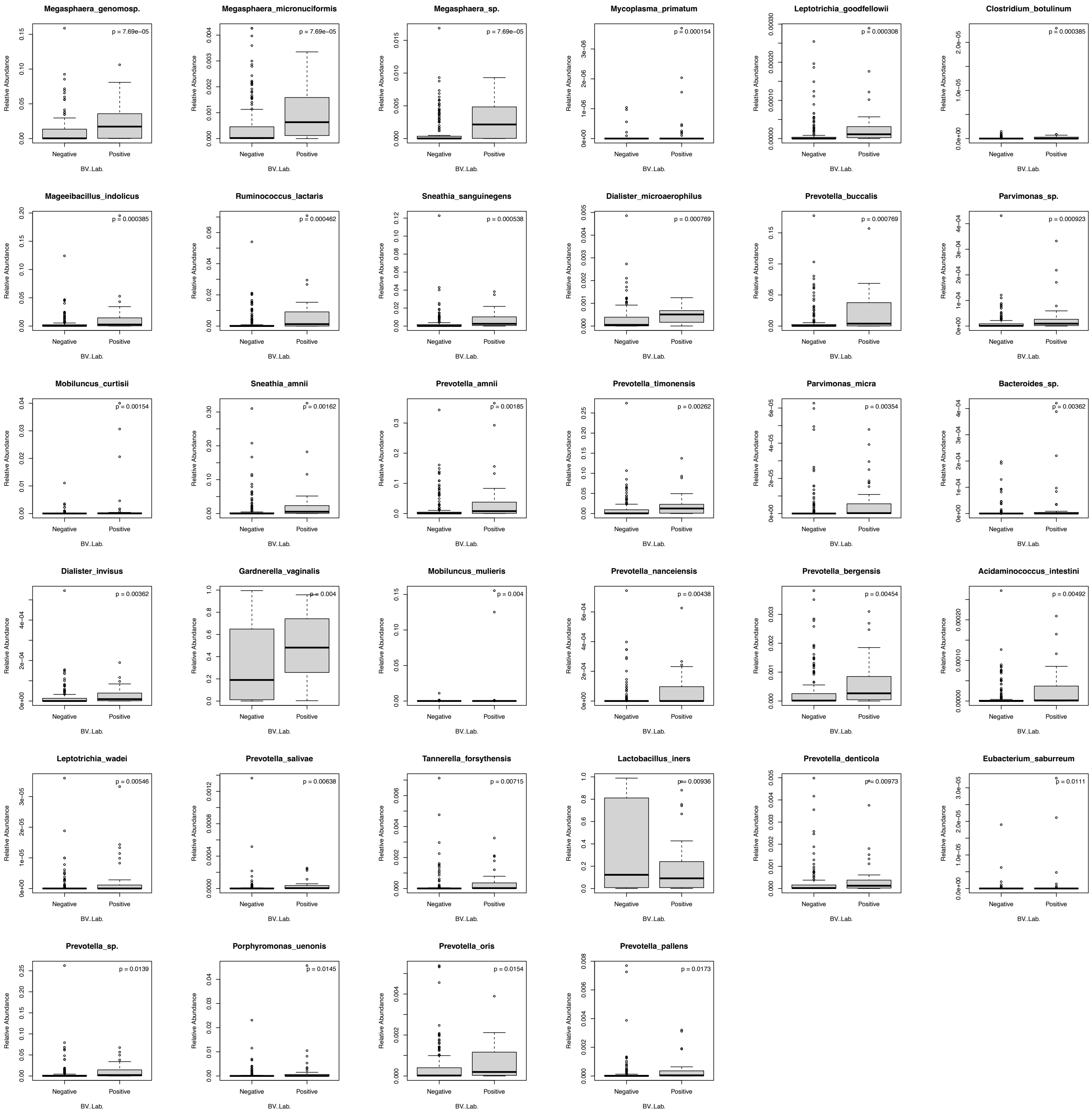

Supplementary Figure 3B

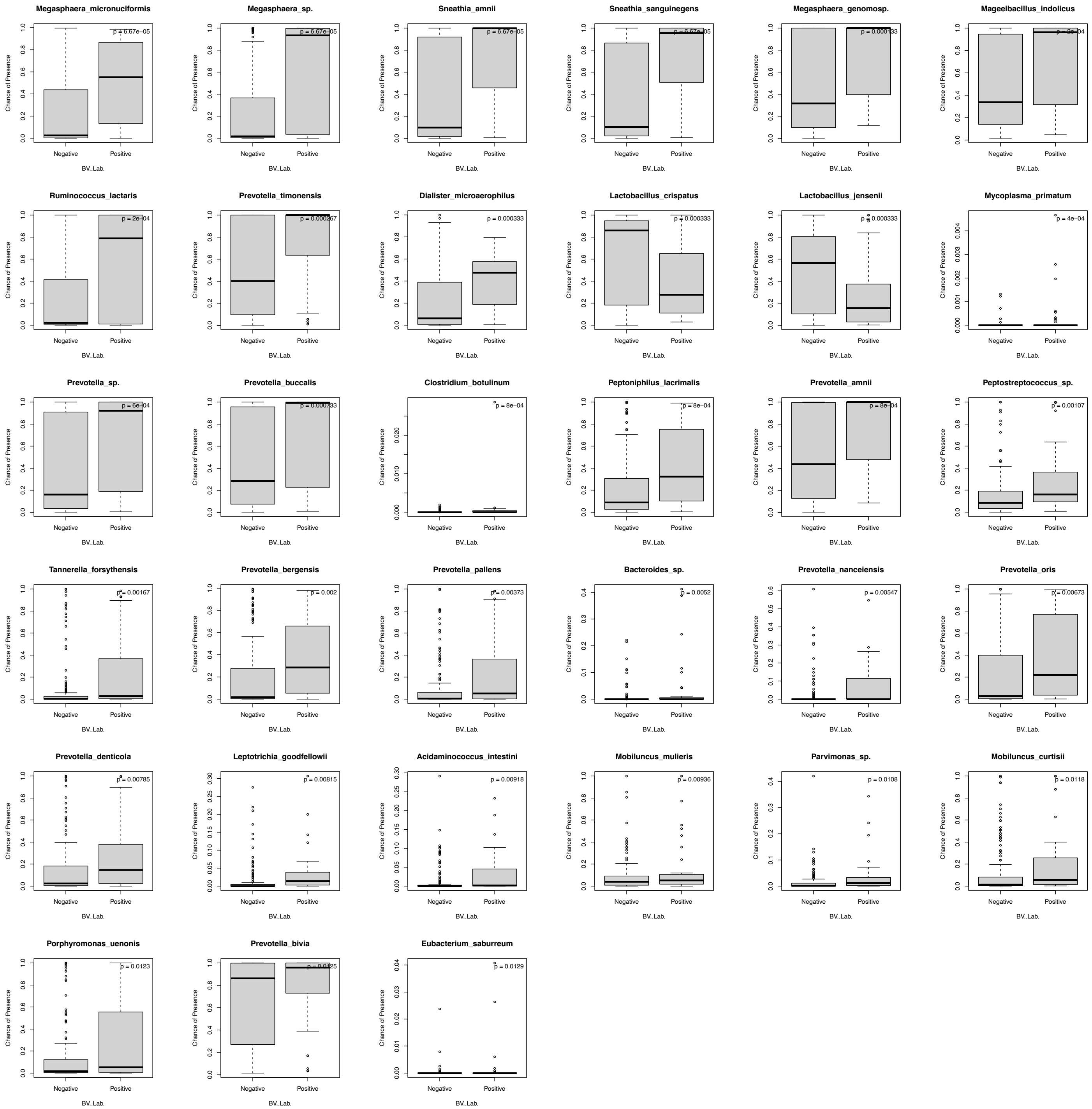

Supplementary Figure 4

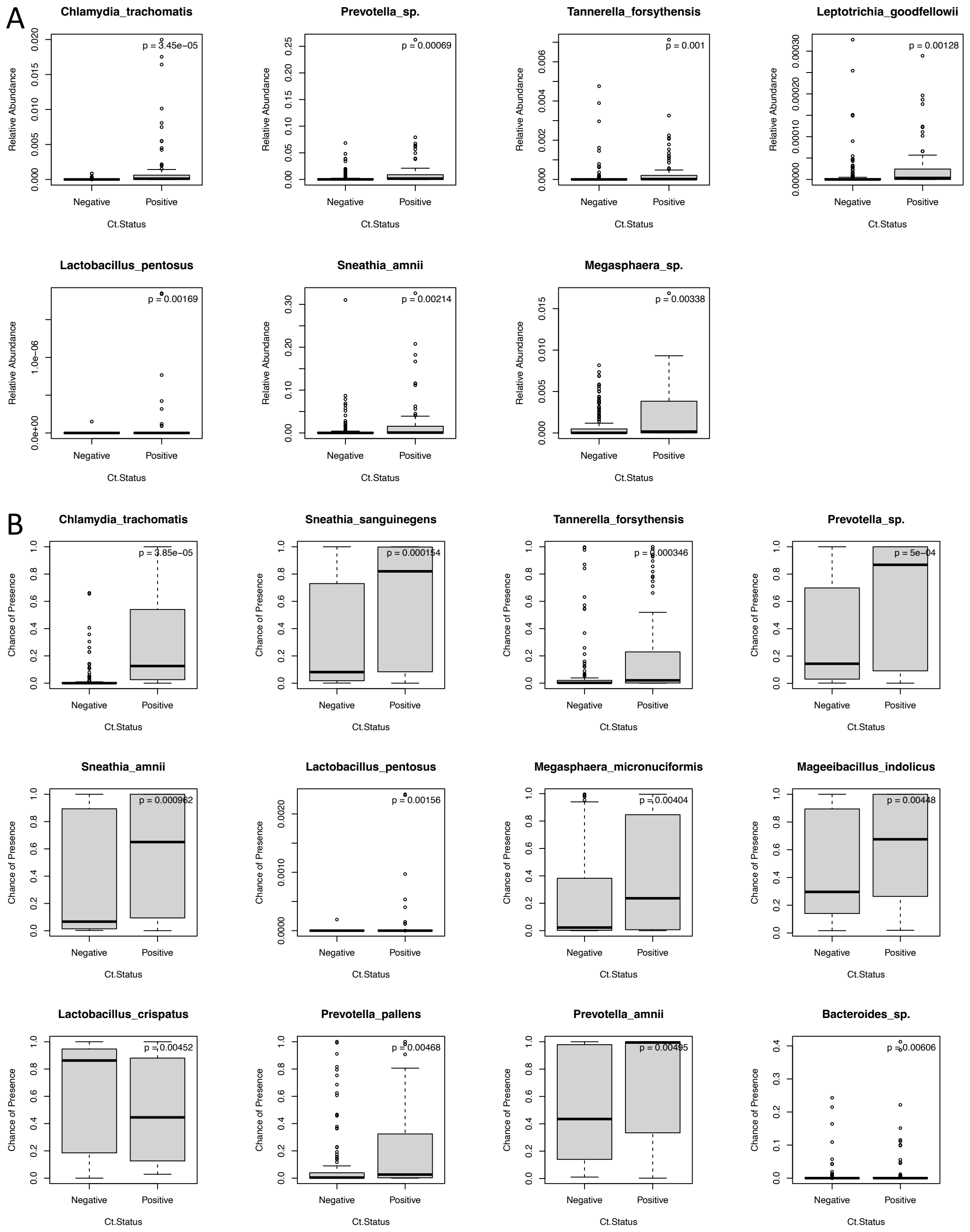

Supplementary Figure 5

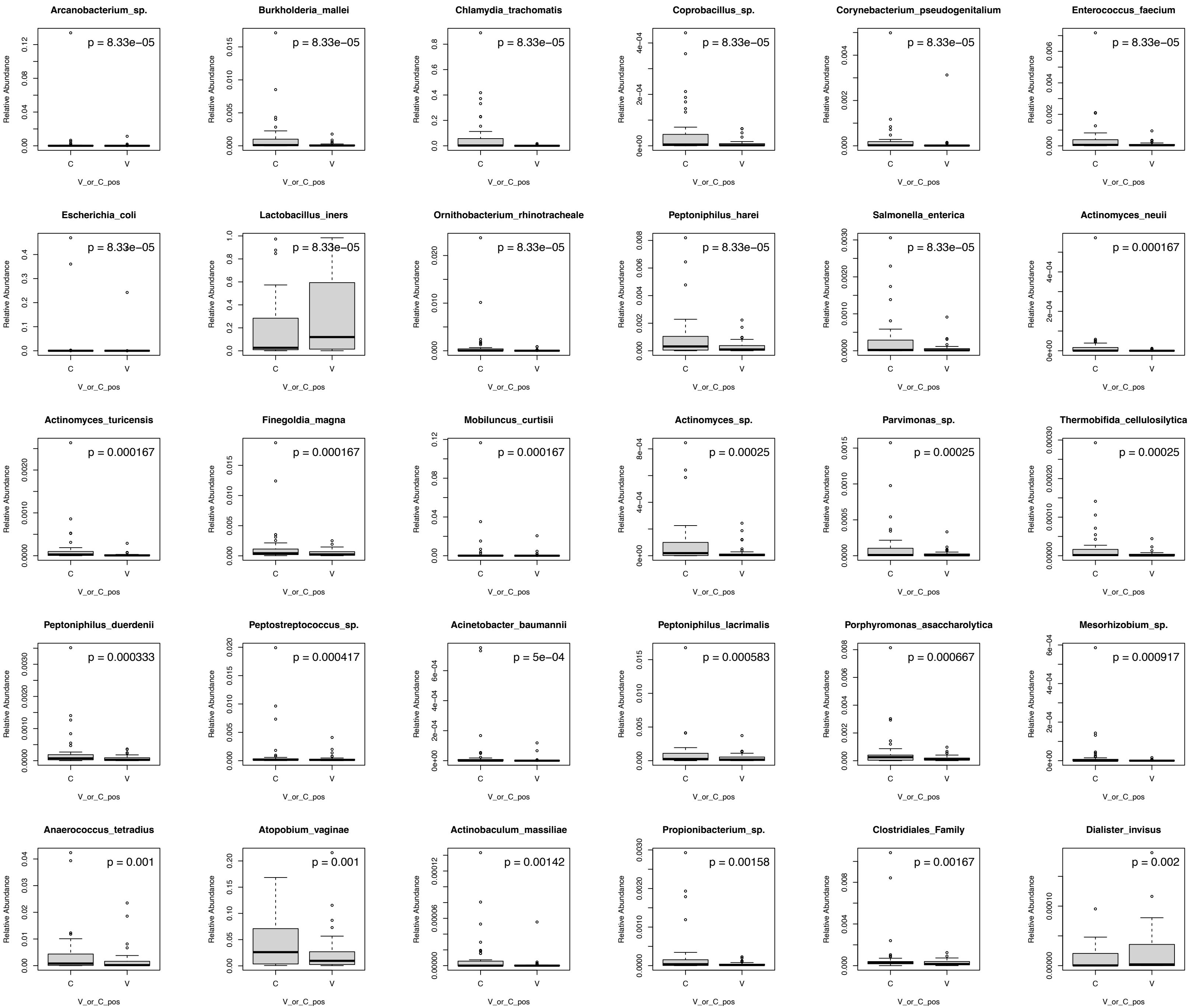

Supplementary Figure 5 (cont.)

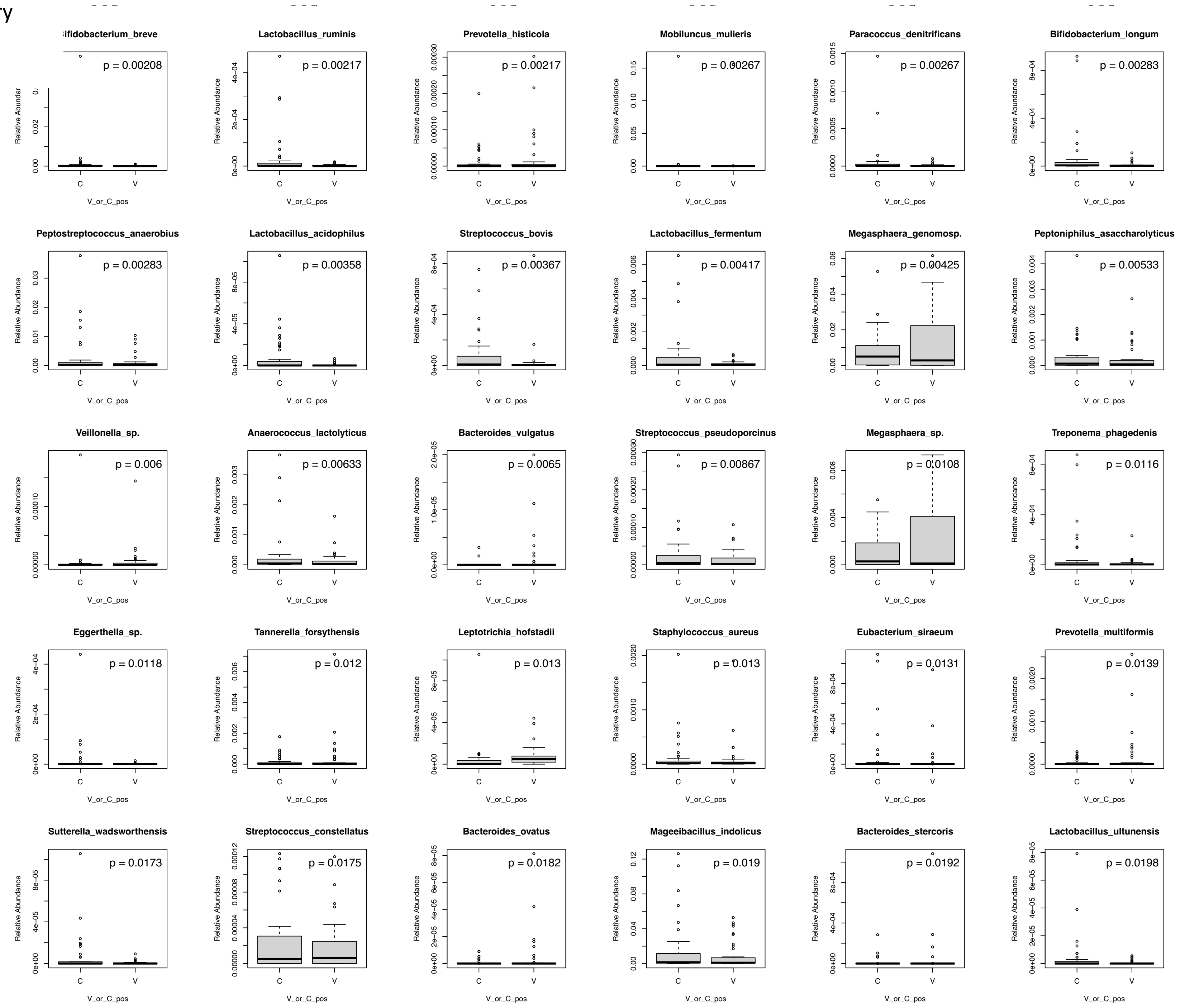

Supplementary Figure 5 (cont.)

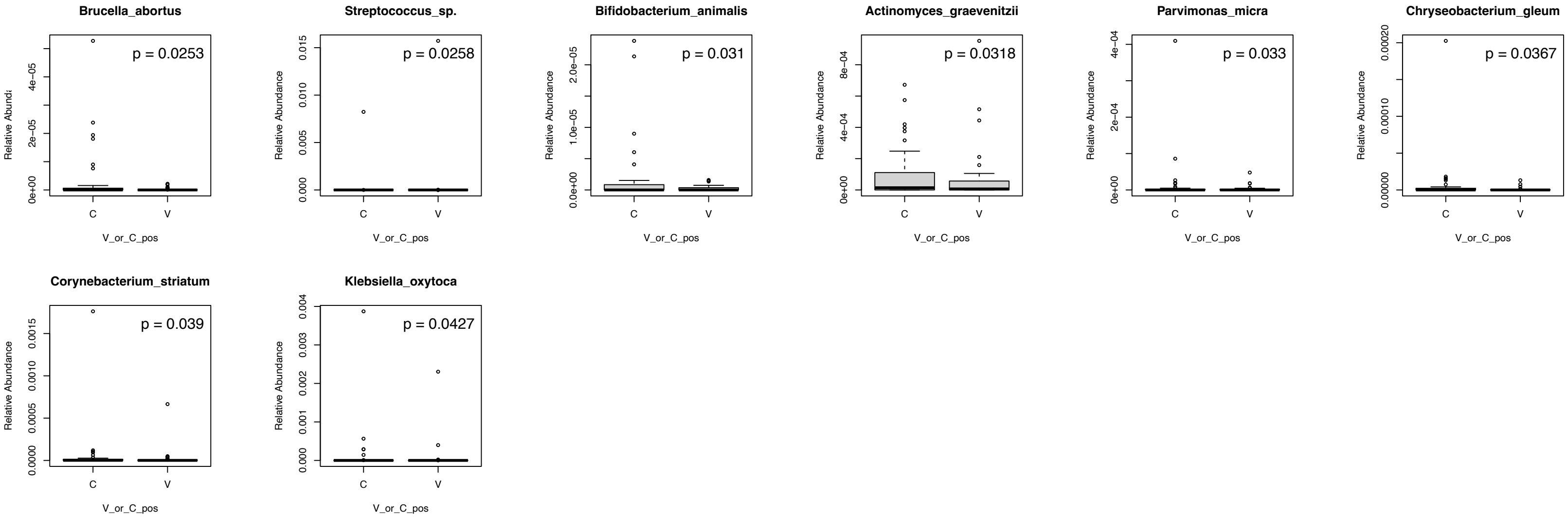

Supplementary Figure 6

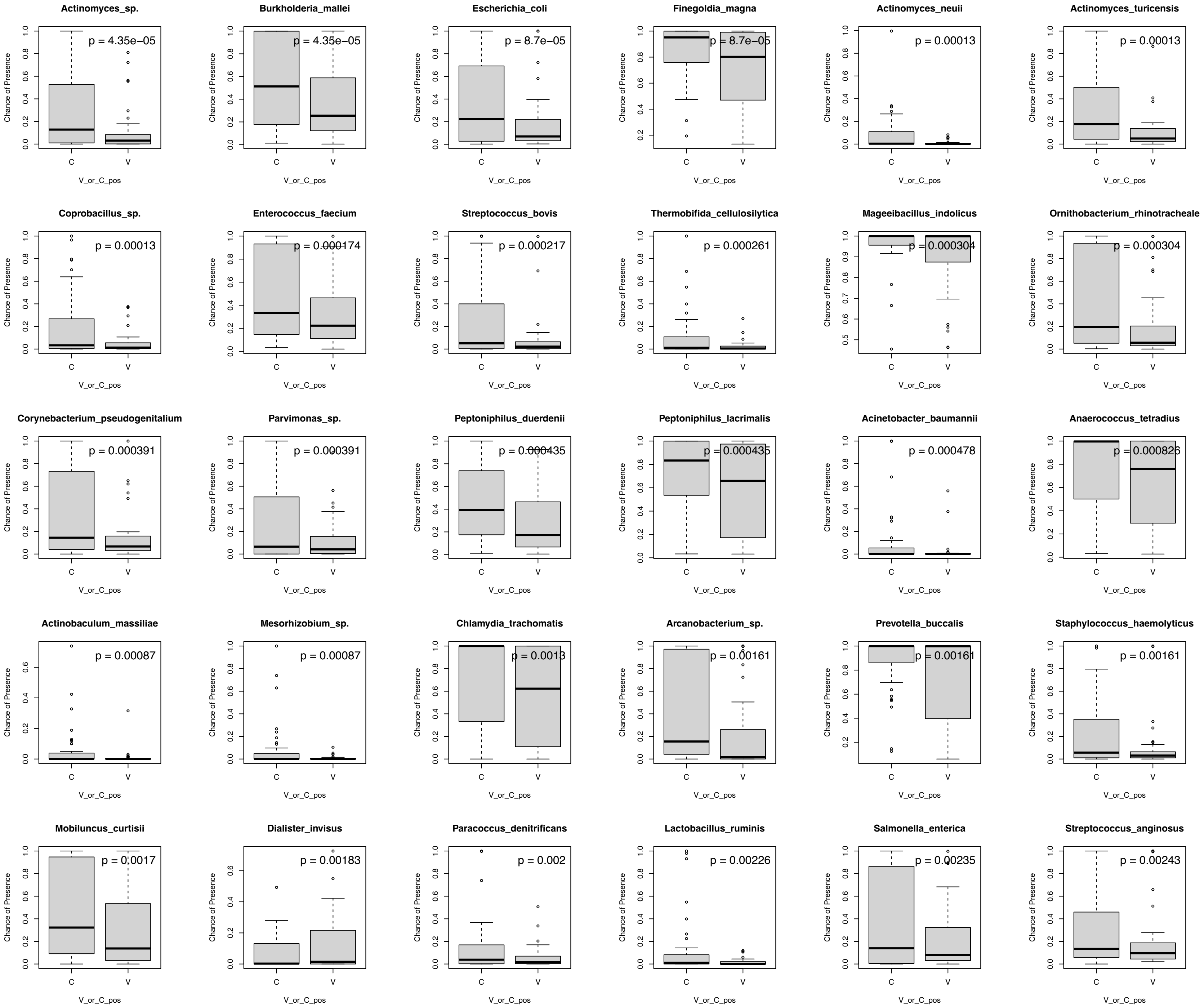

Supplementary Figure 6 (cont.)

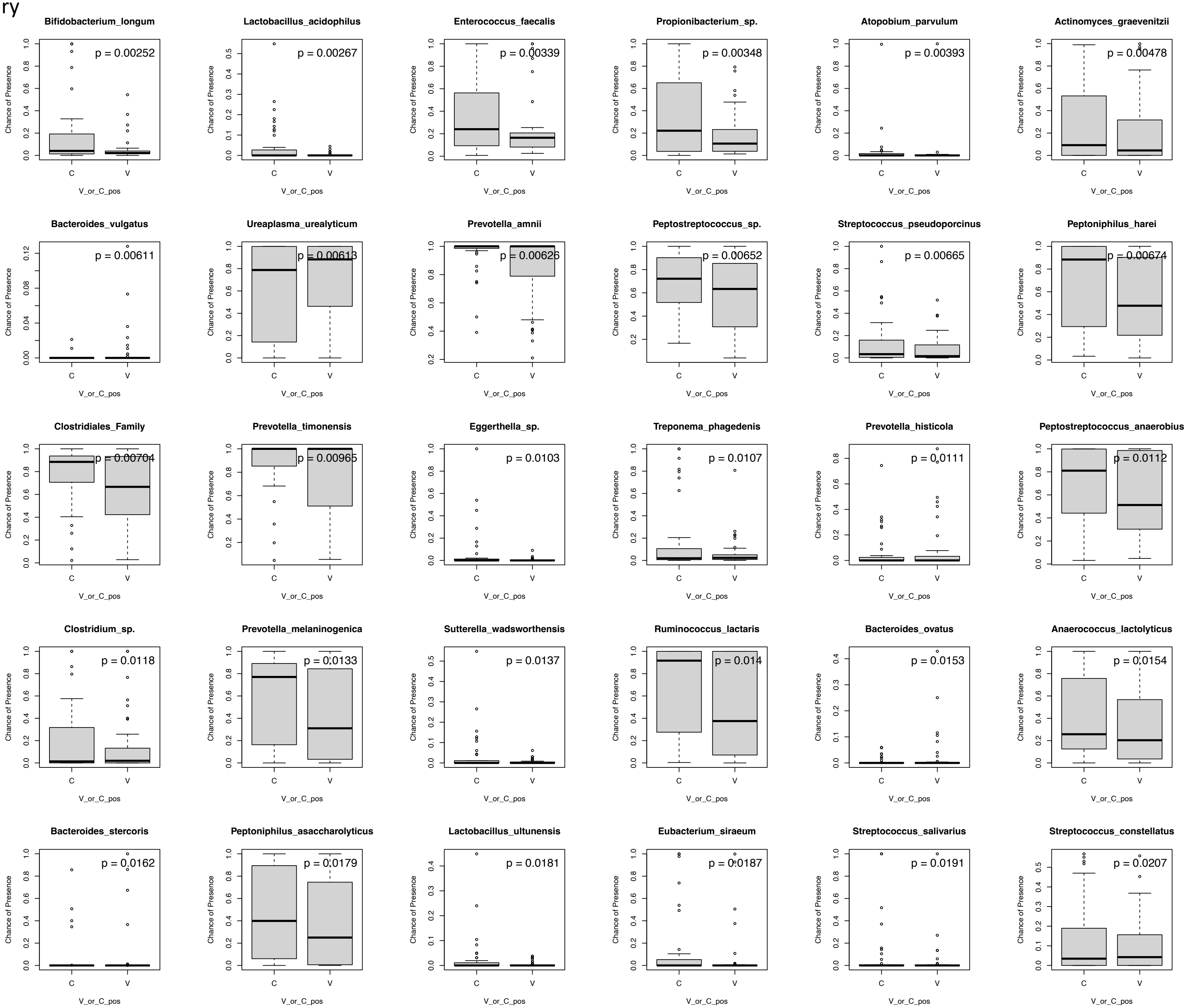

Supplementary Figure 6 (cont.)

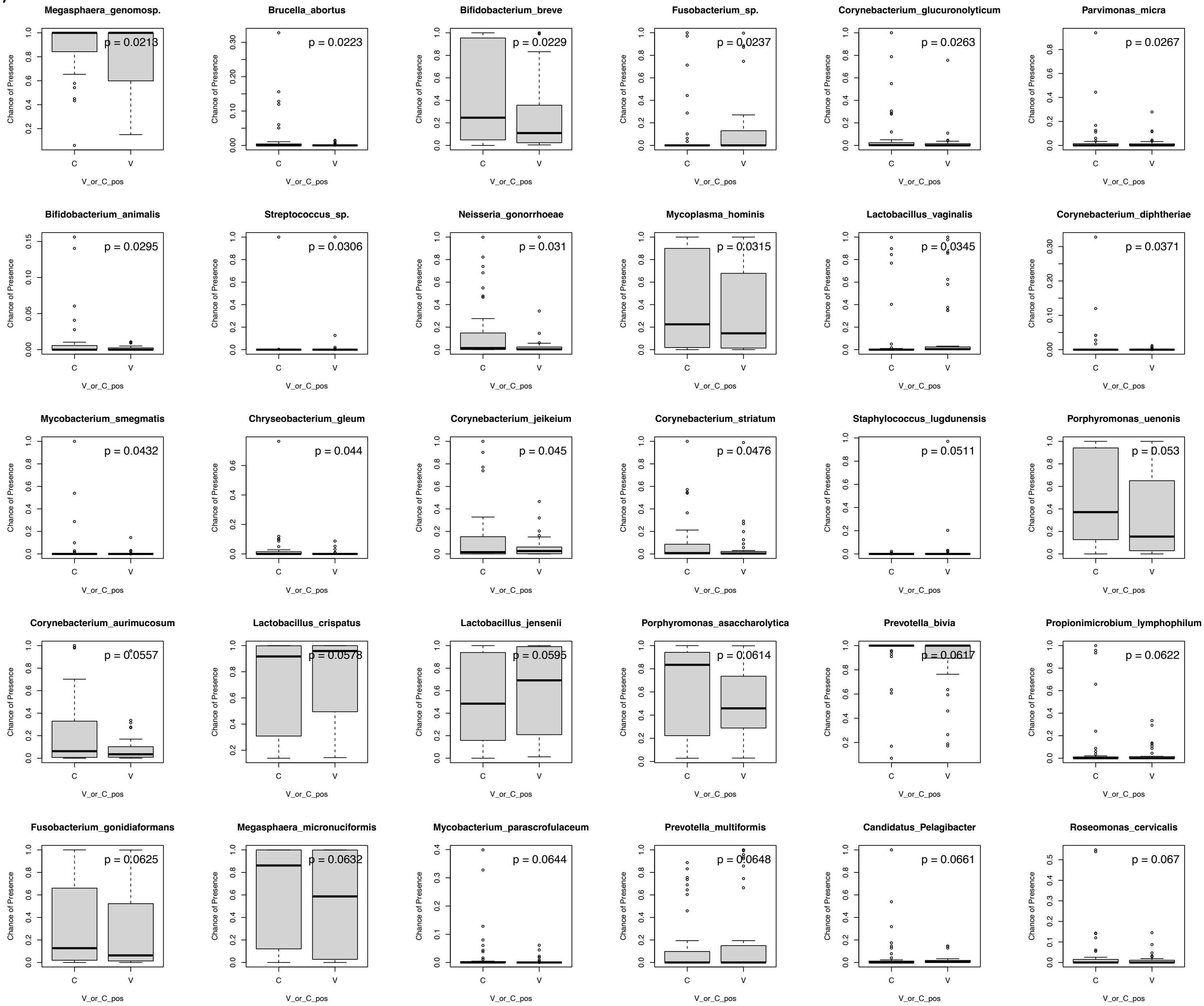

Supplementary Figure 7

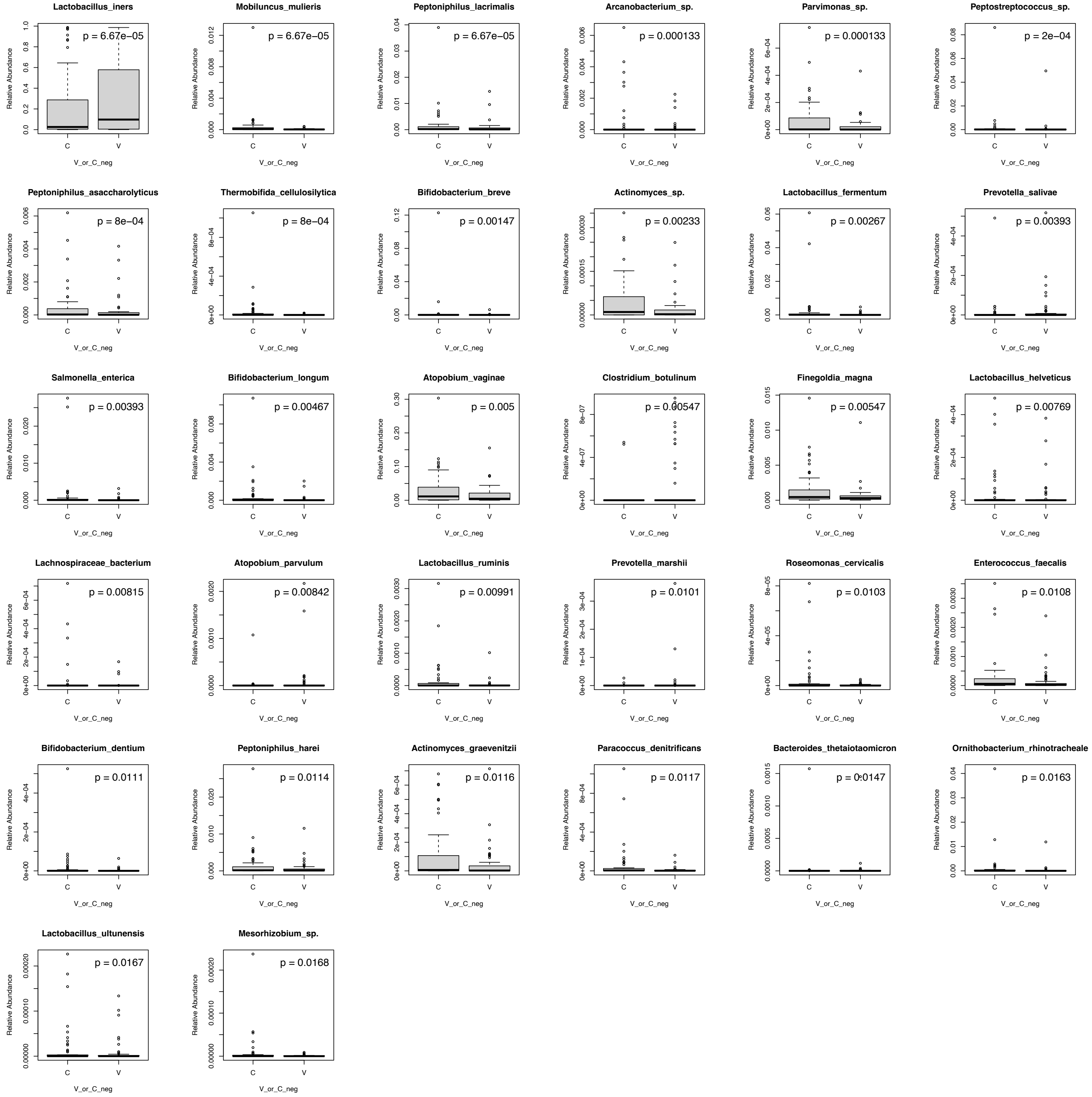

Supplementary Figure 8

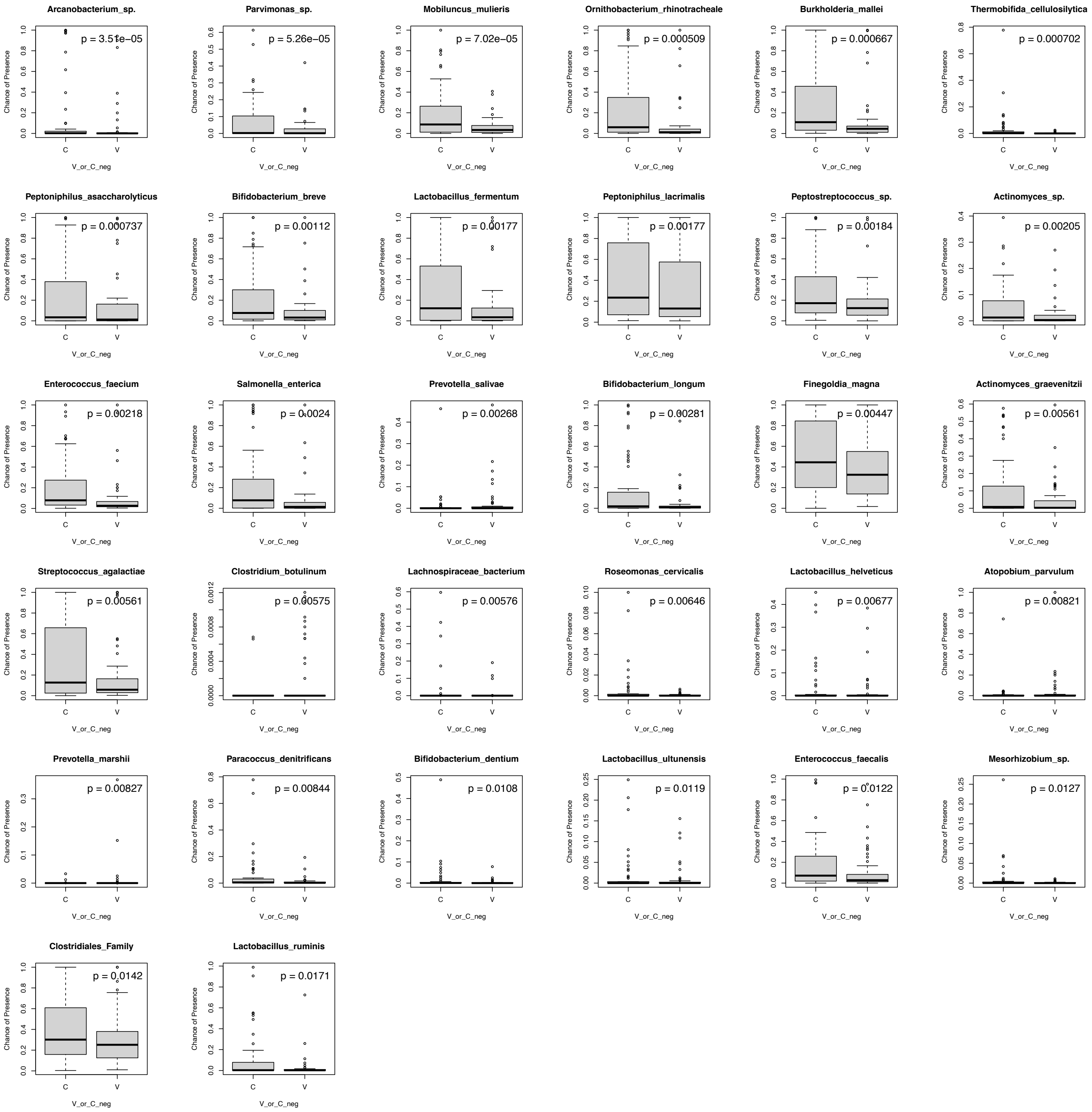

Supplementary Figure 9

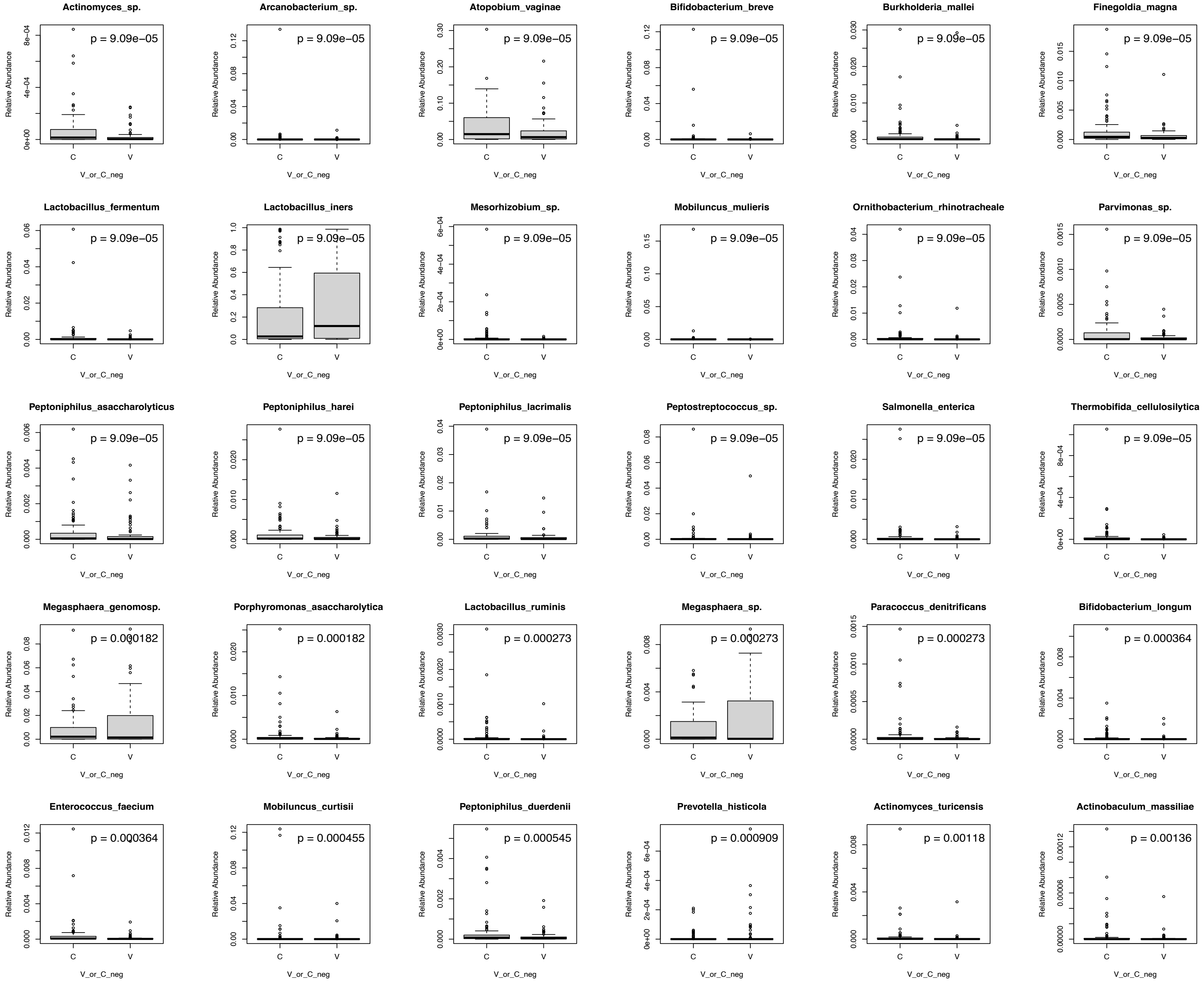

Supplementary Figure 9 (cont.)

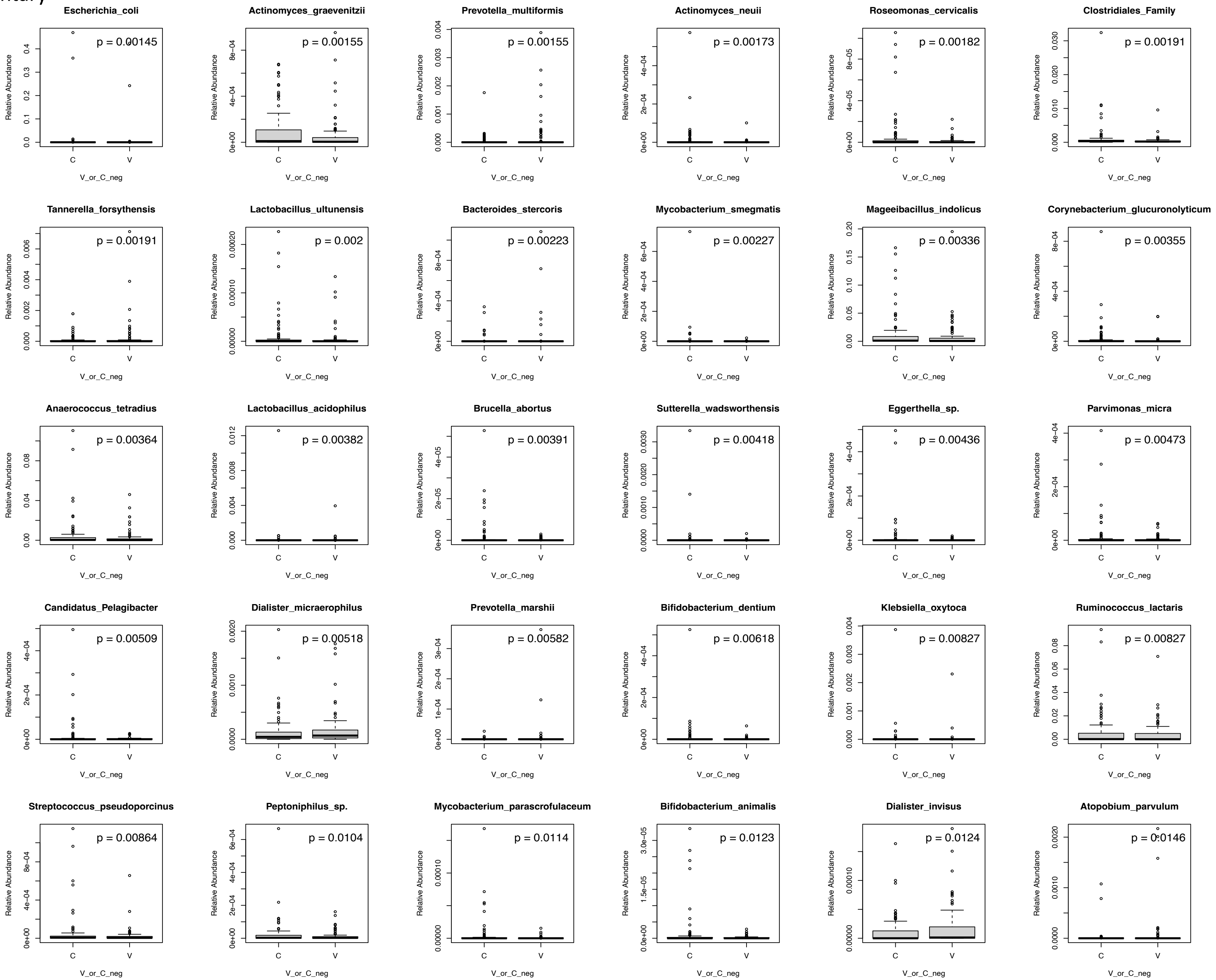

Supplementary Figure 9 (cont.)

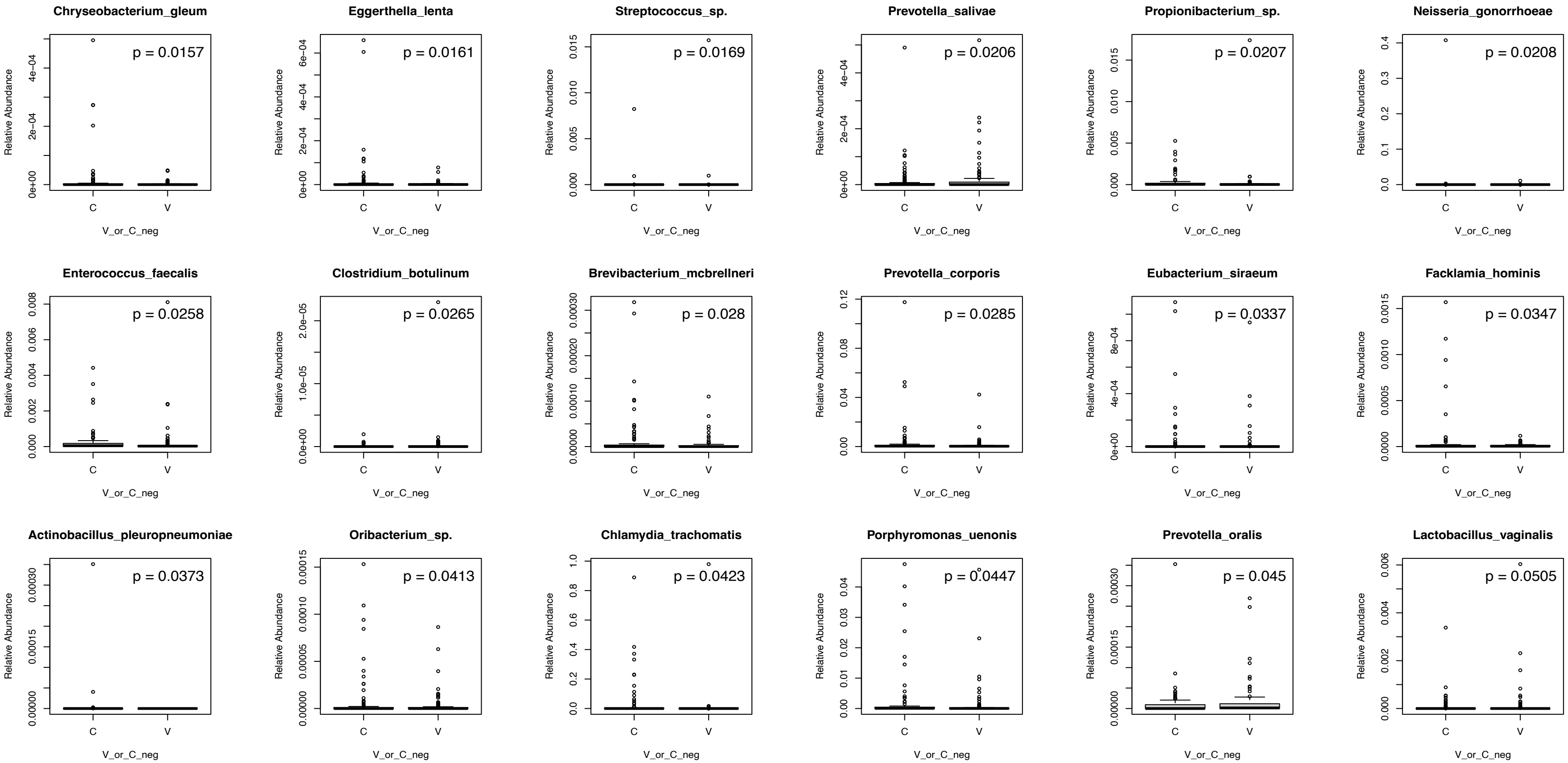

Supplementary Figure 10

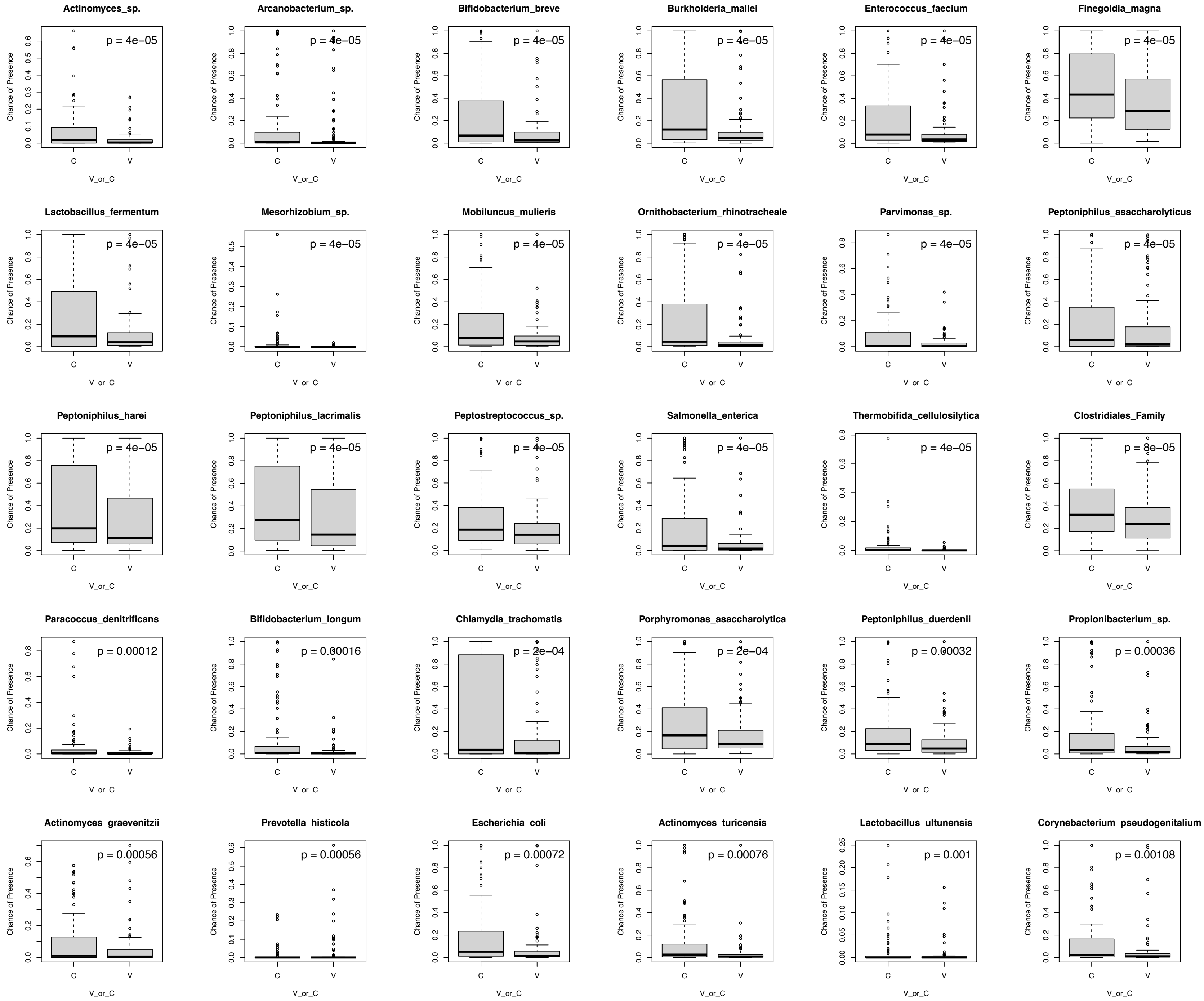

Supplementary Figure 10 (cont.)

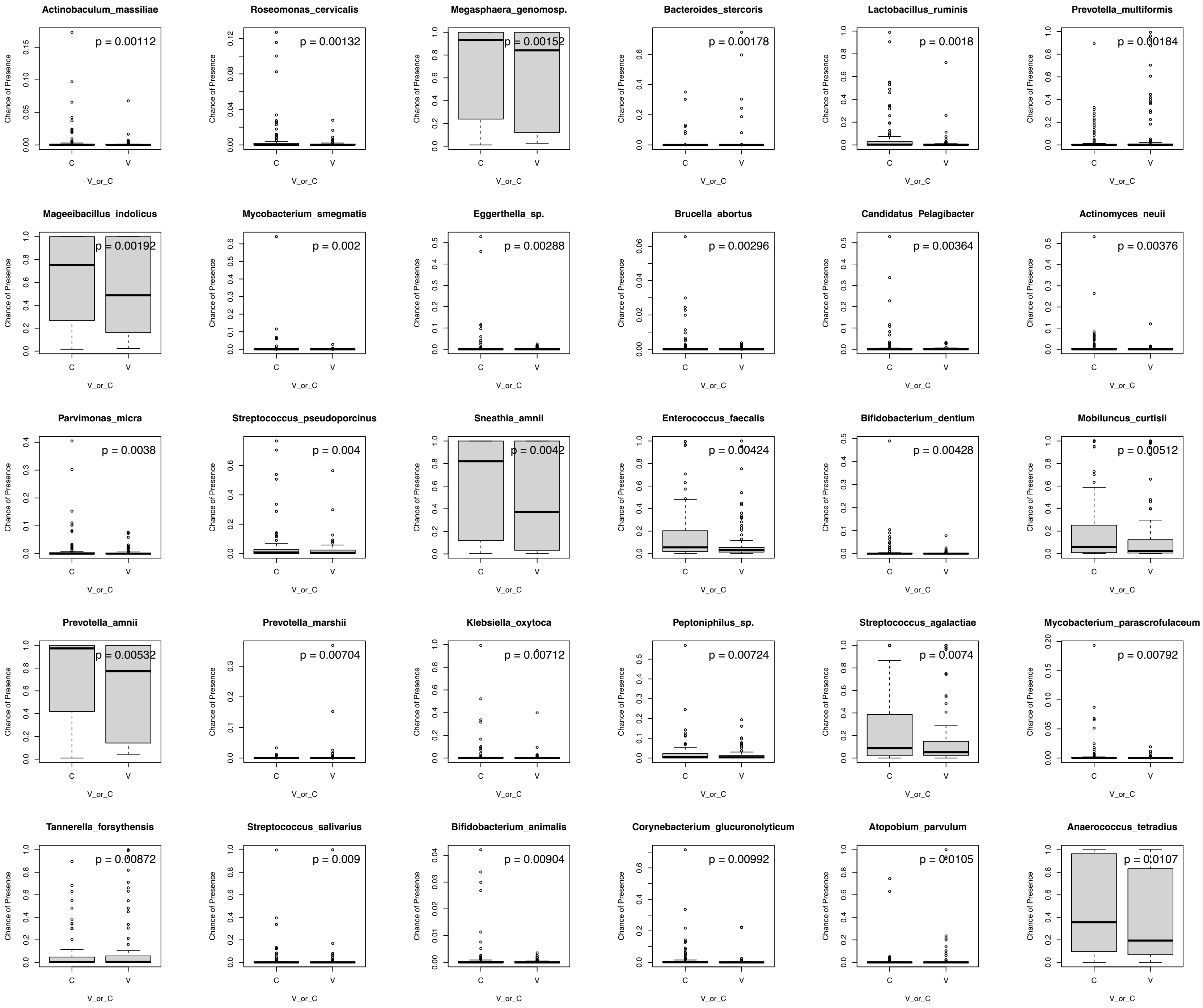

Supplementary Figure 10 (cont.)

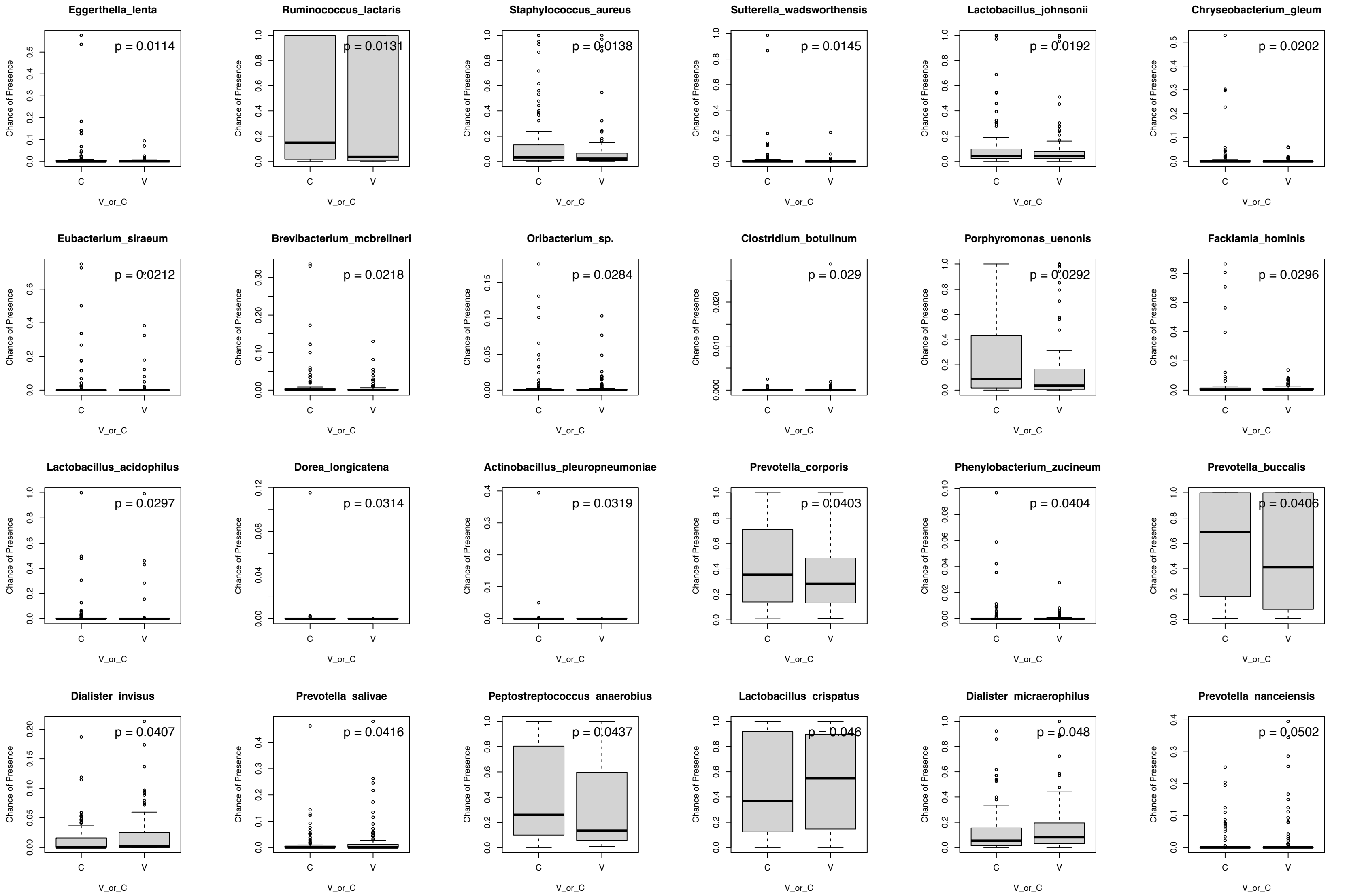

Supplement: Supplemental Figures — Figures S1 to S10. [file mbio.03063-23-s0001.pdf]
